# A Novel Mycovirus Evokes Transcriptional Rewiring in the Fungus *Malassezia* and Stimulates Beta Interferon Production in Macrophages

**DOI:** 10.1128/mBio.01534-20

**Published:** 2020-09-01

**Authors:** Shelly Applen Clancey, Fiorella Ruchti, Salomé LeibundGut-Landmann, Joseph Heitman, Giuseppe Ianiri

**Affiliations:** a Department of Molecular Genetics and Microbiology, Duke University Medical Center, Durham, North Carolina, USA; b Section of Immunology, Vetsuisse Faculty, University of Zurich, Zurich, Switzerland; c Institute of Experimental Immunology, University of Zurich, Zurich, Switzerland; Texas Christian University

**Keywords:** *Malassezia*, skin, beta interferon, mycoviruses

## Abstract

Mycoviruses infect fungi, and while most persist asymptomatically, there are examples of mycoviruses having both beneficial and detrimental effects on their host. Virus-infected *Saccharomyces* and *Ustilago* strains exhibit a killer phenotype conferring a growth advantage over uninfected strains and other competing yeast species, whereas hypovirus-infected Cryphonectria parasitica displays defects in growth, sporulation, and virulence. In this study, we identify a double-stranded RNA (dsRNA) mycovirus in five *Malassezia* species. Sequence analysis reveals it to be a totivirus with two dsRNA segments: a larger 4.5-kb segment with genes encoding components for viral replication and maintenance, and a smaller 1.4-kb segment encoding a novel protein. Furthermore, transcriptome sequencing (RNA-seq) of virus-infected versus virus-cured Malassezia sympodialis revealed an upregulation of dozens of ribosomal components in the cell, suggesting the virus modifies the transcriptional and translational landscapes of the cell. Given that *Malassezia* is the most abundant fungus on human skin, we assessed the impact of the mycovirus in a murine epicutaneous infection model. Although infection with virus-infected strains was not associated with an increased inflammatory response, we did observe enhanced skin colonization in one of two virus-infected *M. sympodialis* strains. Noteworthy, beta interferon expression was significantly upregulated in bone marrow-derived macrophages when challenged with virus-infected, compared to virus-cured, *M. sympodialis*, suggesting that the presence of the virus can induce an immunological response. Although many recent studies have illuminated how widespread mycoviruses are, there are relatively few in-depth studies about their impact on disease caused by the host fungus. We describe here a novel mycovirus in *Malassezia* and its possible implications in pathogenicity.

## Abstract (precis)

*Malassezia* species represent the most common fungal inhabitant of the mammalian skin microbiome and are natural skin commensal flora. However, these fungi are also associated with a variety of clinical skin disorders. Recent studies have reported associations of *Malassezia* with Crohn’s disease and pancreatic cancer, further implicating this fungal genus in inflammatory and neoplastic disease states. Because *M. sympodialis* has lost genes involved in RNA interference (RNAi), we hypothesized *Malassezia* could harbor dsRNA mycoviruses. Indeed, we identified a novel mycovirus of the totivirus family in several *Malassezia* species and characterized the MsMV1 mycovirus of *M. sympodialis*. We found conditions that lead to curing of the virus and analyzed isogenic virus-infected/virus-cured strains to determine MsMV1 genetic and pathogenic impacts. MsMV1 induces a strong overexpression of transcription factors and ribosomal genes, while downregulating cellular metabolism. Moreover, MsMV1 induced a significantly higher level of beta interferon expression in cultured macrophages. This study sheds light on the mechanisms of pathogenicity of *Malassezia*, focusing on a previously unidentified novel mycovirus.

## INTRODUCTION

Viruses are widely distributed in nature and are found in a variety of hosts, ranging from single-celled organisms to plants and mammals. The presence of mycoviruses, viruses that infect fungi, can be benign but is also associated with toxin secretion in yeast, hypovirulence in pathogenic plant fungi, and hypervirulence in dimorphic fungi (1–5). Mycoviruses are typically composed of double-stranded RNA (dsRNA) genomes, but positive-sense and negative-sense single-stranded RNA viruses, as well as circular single-stranded DNA (ssDNA) viruses, have been reported to infect fungi (6). Overall, more than 10 different families of viruses have been isolated from fungal hosts (7). Despite similar genomic features shared between mycoviruses and human viruses from the same family, one clear difference between the two is the absence of cell-to-cell transmission proteins in mycoviruses. Therefore, mycoviruses typically lack an extracellular stage and are transmitted only during cell division, or by cell or hyphal fusion (8–10).

A well-known example of a mycovirus is the killer virus of Saccharomyces cerevisiae. This toxin-producing dsRNA virus was characterized in the 1970s (11) and found to have two dsRNA segments: a larger, 4.5-kb dsRNA and a smaller, 1.5-kb satellite dsRNA. The large segment, called L-A, is required for viral replication and maintenance. The smaller segment, called M, encodes a preprotoxin, which is processed into a mature toxin by host enzymes and then secreted from the cell (12, 13). During the replication cycle of L-A, the single-stranded positive-strand [(+)ssRNA] is transcribed by the virally encoded replication machinery and released from the virion to serve two roles: (i) it is the mRNA template for translation of the Gag and Gag-Pol fusion protein, and (ii) it is the template that is packaged into new viral particles where transcription reoccurs (13, 14). Yeast cells harboring both the L-A and M dsRNA segments display a growth advantage over strains lacking these components. When toxin-producing strains compete with wild-type strains, the virus-infected strains secrete the mature killer toxin killing neighboring cells, whereas the toxin-producing cells remain unharmed (15). A similar fitness advantage is conferred by toxin-producing strains of Ustilago maydis (2).

In contrast to the killer viruses that provide virus-infected strains a growth advantage, the mycovirus that infects Cryphonectria parasitica, the causative agent of chestnut blight (which decimated the population of chestnut trees in the United States in the early 1900s by killing more than 1 billion trees), impairs growth of the fungus (16, 17). The growth defect of virus-infected strains results in reduced virulence, hence the name *Cryphonectria parasitica* hypovirus (CHV). Five different viral segments isolated from *C. parasitica* have been shown to confer a high level of hypovirulence, although the level of impact on fungal growth observed in the infected fungal strain appears to be virus strain dependent (16, 18). A mycovirus infecting the filamentous fungus Pseudogymnoascus destructans has also been implicated in the epidemiology of white-nose syndrome, a lethal fungal infection in bats, as bats in North America have experienced 5 million deaths in the past decade, and all *P. destructans* isolates from bats in this region harbor a mycovirus (19). This is compared to European and Asian *P. destructans* isolates, which do not harbor the mycovirus, and the resident bat populations appear to be resistant to the endemic fungus.

RNA interference (RNAi) is an important pathway conserved in many organisms and serves to regulate gene expression, suppress transposon movement, and maintain genome integrity. Additionally, a functional RNAi pathway has been shown to suppress mycovirus proliferation (20, 21). The biogenesis of small RNAs (sRNAs) originating from the infecting virus (termed virus-derived small interfering RNAs [vsiRNAs]) is analogous to that of endogenous sRNAs, requiring essential components such as dicer (*DCL*), argonaute (*AGO*), and RNA-dependent RNA polymerase (*RDP*) (22). It was previously thought that mycoviruses might survive only in species that lack a functional RNAi pathway as introduction of argonaute and dicer into yeast resulted in the elimination of the mycovirus (20). To this end, fungal species lacking canonical RNAi pathways have been good candidates in which to screen for mycoviruses. However, increased research on mycoviruses over the past decade has produced reports showing that mycoviruses have developed strategies to counteract the RNAi defense mechanisms that suppress antiviral activity (5). In Aspergillus nidulans, infection of conidia with Virus 1816 suppresses inverted repeat transgene (IRT)-induced RNA silencing, and when the virus was cleared from ascospores, IRT silencing was restored (23). Other examples of mycovirus-induced RNAi suppression in *C. parasitica* and Rosellinia necatrix involve viral targeting of the RNAi machinery. In the case of *C. parasitica*, CHV1-EP713 encodes a papain-like protease (p29) that suppresses hairpin RNA-triggered silencing in the fungus (24). In *R. necatrix*, the mycoreovirus RnMyRV3 s10 gene encodes a product that appears to suppress RNA silencing by interfering with the dicing of dsRNA (25). Most recently, a novel partitivirus, TmPV1, was identified in Talaromyces marneffei and is correlated with the downregulation of the *dcl*-1, *dcl*-2, and *qde*-2 RNAi genes in both virus-infected yeast and mycelia (4). Examples of virus-induced RNAi suppression spanning multiple classes of fungi are evidence that RNAi functions to control viral levels and that mycoviruses have evolved ways to counteract this regulation.

The genus *Malassezia* is a monophyletic group of lipophilic yeasts that colonize sebaceous skin sites and represent more than 90% of the skin mycobiome. Despite being a commensal yeast, *Malassezia* is associated with a number of common skin disorders, such as seborrheic dermatitis, atopic eczema, and pityriasis versicolor, and also systemic infections (26). Recently, two landmark studies reported the association of *Malassezia* with gastrointestinal disorders such as Crohn’s disease in a subset of patients with CARD9 mutations (which enhance sensitivity to fungal pathogen-associated molecular patterns [PAMPs]) and pancreatic cancer (27, 28), highlighting the importance of this understudied fungal genus as a crucial component of the human mycobiome. All *Malassezia* species lack the fatty acid synthase gene and thus are lipid dependent, relying on exogenous fatty acids for growth. This feature makes *Malassezia* unique and fastidious to study under laboratory conditions, and as a consequence relatively limited research has been done on the basic biology of this organism.

To date, 18 different species of *Malassezia* have been identified and sequence for 15 of these genomes is publicly available. Of these, the Malassezia sympodialis ATCC 42132 genome is the best-annotated reference genome based on whole-genome sequencing, transcriptome sequencing (RNA-seq), and proteomics (29, 30). As commensal inhabitants of the skin, *Malassezia* yeasts interact with the skin immune system, and specific T cells, as well as IgG and IgM antibodies, can be detected in healthy individuals, whereas specific IgE antibodies are prevalent in a large proportion of patients suffering from atopic eczema. A number of molecules with immunoglobulin E (IgE)-binding capabilities have been identified in extracts of *Malassezia* and have been named MalaS allergens. Ten allergens have been identified in *M. sympodialis*, and three in M. furfur. Eight of these allergens are conserved proteins that share high similarity with the corresponding mammalian homolog, suggesting that the induction of autoreactive T cells by *Malassezia* allergens could play a role in sustained inflammation (26, 31). Nevertheless, both the fungal and host factors contributing to the transition of *Malassezia* from commensal to pathogen are unknown, and this is an active area of research.

*Malassezia* has a relatively small genome of 7.7 Mb and due to its reduced size has lost nearly 800 genes compared to other sequenced Basidiomycota, including genes of the RNAi pathway (29). Given that *Malassezia* has lost the RNAi machinery, we hypothesized it could harbor mycoviruses and screened our collection of *Malassezia* strains for novel dsRNA viruses. Here, we report the discovery of a novel mycovirus from *M. sympodialis*, MsMV1. Following the screening of three dozen *M. sympodialis* isolates in our collection, we observed that this mycovirus is present in approximately 25% of the isolates. Fungal cells can be cured of the mycovirus upon exposure to high temperature or the antimicrobial agent biphenyl. RNA sequencing of infected and cured strain pairs revealed the complete genome of MsMV1 and illuminated a large shift in the cellular transcriptional profile comparing the congenic virus-infected and virus-cured strains. The presence of MsMV1 correlated with an increased ability to colonize skin in one of two cases tested, and increased production of beta interferon (IFN-β) in cultured macrophages was associated with all virus-infected strains tested. These findings reveal a novel mycovirus harbored by *Malassezia* with possible implications for interactions with the host in both the commensal and disease states.

## RESULTS

### Identification and characterization of a novel mycovirus in *Malassezia*.

**FIG 1:**
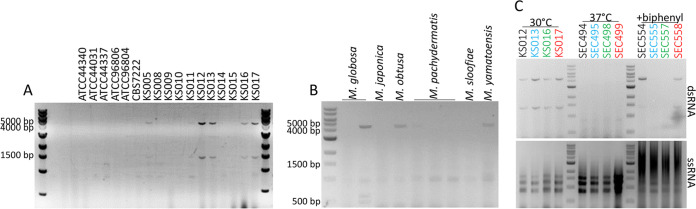
*Malassezia* species harbor dsRNA segments. (A) dsRNA from representative *M. sympodialis* strains. (B) dsRNA from additional *Malassezia* species. Strain names can be found in Table 1. (C) dsRNA (upper panel) and ssRNA (lower panel) from *M. sympodialis* strains passaged at 30°C, at 37°C, and on mDixon medium containing 10 μg/ml biphenyl. All dsRNA and ssRNA extracts were visualized by electrophoresis in 0.8% agarose gels with 0.5 μg/ml ethidium bromide.

Whole-genome sequencing revealed that *Malassezia* species have lost the genes required for a functional RNAi pathway (29); therefore, we hypothesized the fungus had the capacity to harbor mycoviruses. Following the screening of *M. sympodialis*, M. pachydermatis, and *M. furfur* laboratory freezer stocks, dsRNA species were detected in 11 of 36 *M. sympodialis* isolates (30.5%), one of three *M. pachydermatis* isolates (33.3%), and no *M. furfur* isolates. dsRNA species were also found in one of two M. globosa isolates, one M. obtusa isolate, and one M. yamatoensis isolate (Fig. 1A and B and Table 1; see also Fig. S1 in the supplemental material). For *M. sympodialis*, two dsRNA segments were visualized on an ethidium bromide-stained agarose gel, one at ∼4.5 kb and another at ∼1.4 kb. The sizes of these dsRNA segments are similar to those found in S. cerevisiae strains that produce killer toxins (1), suggesting the segments may correspond to a helper virus and a satellite RNA encoding a toxin precursor. *M. obtusa* and *M. yamatoensis* appear to have only the larger ∼4.5-kb species as no dsRNA species of ∼1.4 kb was detected. *M. globosa* also has an ∼4.5-kb dsRNA species as well as two smaller species of ∼500 bp and ∼600 bp (Fig. 1B).

**TABLE 1:** Strains used in dsRNA gel images^*a*^

Species	Strain	dsRNA	Fragment size(s) (bp)	Clinical data
*M. sympodialis*	ATCC 44340	N		U
	ATCC 44031	N		TV
	ATCC 44337	N		U
	ATCC 96806	N		H
	ATCC 96804	N		TV
	CBS7222	N		U, ear
	KS005	Y	1,500, 4,500	H
	KS008	N		H
	KS009	N		H
	KS010	N		H
	KS011	N		H
	KS012	Y	1,500, 4,500	H
	KS013	Y	1,500, 4,500	H
	KS014	N		H
	KS015	N		H
	KS016	Y	1,500, 4,500	H
	KS017	Y	1,500, 4,500	H
	ATCC 42132	N		TV
	ATCC 44341	N		H
	KS004	N		H
	KS020	N		H
	KS022	N		H
	KS024	Y	1,500, 4,500	H
	KS025	Y	1,500, 4,500	H
	KS026	N		H
	KS027	N	1,500, 4,500	H
	KS028	Y	1,300, 45,00	H
	KS029small	N		H
	KS029big	N		H
	KS030	N		H
	KS031	N		H
	KS032	N		H
	NIH70	N		H
	NIH71	N		H
	NIH90	Y	4,500	H
*M. furfur*	CBS1878a	N		D, scalp
	CBS1878b	N		D, scalp
	CBS1878c	N		D, scalp
	CBS4172a	N		U
	CBS4172b	N		U
	CBS7019a	N		PV, torso
	CBS7019b	N		PV, torso
	CBS7019c	N		PV, torso
	CBS7710a	N		U
	CBS7710b	N		U
	CBS7982a	N		H, ear
	CBS7982b	N		H, ear
	JPLK23	N		U, blood
	WL_MF1	N		U, scalp
*M. globosa*	CBS7866	N		U
	CBS7874	Y	500, 600, 4,500	D, scalp
*M. japonica*	CBS9431	N		H
*M. obtusa*	CBS7876	Y	4,500	U
*M. pachydermatis*	DUMC184.03	Y	4,500	U*, ear
	CBS1879a	N		OE*, ear
	CBS1879b	N		OE*, ear
*Malassezia slooffiae*	CBS7956	N		H*, ear
*M. yamatoensis*	CBS9725	Y	4,500	SD

a Strains are listed top to bottom in order of gel wells from left to right. If known, clinical data and sample location are listed. U, unknown; H, healthy individual; D, dandruff; TV, tinea versicolor; PV, pityriasis versicolor; SD, seborrheic dermatitis; OE, otitis externa. * indicates strains sampled from an animal.

10.1128/mBio.01534-20.4 FIG S1 *Malassezia* species harbor dsRNA segments. dsRNA from select *M. sympodialis* (A) and *M. furfur* (B) strains was analyzed by electrophoresis on a 0.8% agarose gel with 0.5 μg/ml ethidium bromide. Download FIG S1, TIF file, 0.5 MB. Copyright © 2020 Clancey et al. 2020 Clancey et al. This content is distributed under the terms of the Creative Commons Attribution 4.0 International license.

In the present study, we focused on the characterization of the mycovirus of *M. sympodialis* as a representative species of the *Malassezia* genus. To unveil the biological and physiological roles of the putative *Malassezia* mycovirus, a crucial first step was to define conditions that allow elimination (i.e., curing) of the mycovirus. In other fungi, mycoviruses can be cured by exposure to stressful conditions or certain drugs. In S. cerevisiae, exposure to the fungicide cycloheximide cures killer cells of the killer phenotype (9). This has various rates of success in other organisms, such as *Aspergillus*, where a less than 50% success rate of mycovirus curing was observed after exposure to cycloheximide (32). In filamentous fungi, conidial isolation and hyphal tipping are more commonly used methods for curing (32), and polyethylene glycol was shown to cure *P. destructans* of PdPV-pa infection (19). To determine if *M. sympodialis* could be cured of the dsRNA, the strains were passaged on modified Dixon’s (mDixon) medium and subjected to various stresses. We found that six passages on mDixon medium with no additives at high temperature (37°C) resulted in a complete loss of both dsRNA segments in four *M. sympodialis* virus-infected strains (Fig. 1C, middle section). Following a single passage on mDixon medium with 10 μg/ml biphenyl, strains SEC555 and SEC557 derived from KS013 and KS016, respectively, lost both dsRNA species, while strains SEC554 and SEC558 (derived from KS012 and KS017, respectively) retained both dsRNA species (Fig. 1C, right section). All dsRNA-containing strains retained both segments when passaged on mDixon with no additives at 30°C (Fig. 1C, left section).

To determine if the dsRNA moieties isolated from *M. sympodialis* are of viral origin, the dsRNA molecules of *M. sympodialis* were converted to cDNA and subjected to TA cloning and Sanger sequencing. Clones of the large moiety were obtained from strain KS013, and from KS012 for the smaller one. Primer walking with Sanger sequencing revealed only partial insertions of the two into the cloning plasmid: a 1.3-kb region of the large ∼4.5-kb segment and a 374-bp portion of the ∼1.4-kb segment. BLASTx of the 1.3-kb sequence derived from the larger dsRNA revealed similarities with other mycoviruses deposited in GenBank, while a BLASTx search of a 374-bp sequence derived from the smaller dsRNA found no hit (see below for details). In parallel with the cDNA synthesis, cloning, and sequencing, the congenic virus-infected and virus-cured strains KS012 and SEC494 of *M. sympodialis*, respectively, were subjected to rRNA-depleted RNA sequencing. The rationale of the RNA-seq analysis was 2-fold: first, allowing a more facile identification of the entire viral genome, and second, elucidating possible transcriptomic changes evoked by the mycovirus in the host cells.

For the identification of the viral genome, adapter-free RNA-seq reads derived from *M. sympodialis* virus-infected and virus-cured isolates were assembled with Trinity. The resulting transcripts were searched using the sequences previously obtained through TA cloning as a query, and two perfect hits (E value 0.0) were obtained following a BLASTn search against the virus-infected strain KS012. Both hits are part of two larger independent contigs (DN8321 of 4,613 bp and DN4986 of 4,601 bp; see Text S1 legend for details) that subsequent analyses (described in detail below) reveal represent the entire viral genome. The two identified contigs are identical with the exception of a few nucleotides located at the sequence extremities and outside coding regions, likely residual adapter or assembly artifacts (Text S1). Conversely, BLASTn analysis of the 1.3-kb viral TA-cloned sequence against the transcriptome of the virus-cured isolate SEC494 found only a small 308-bp hit (E value 1e−143) that is part of the *M. sympodialis* ATCC 42132 *MRP4* ortholog, which encodes a mitochondrial ribosomal protein of the small subunit (MSYG_4110, accession no. SHO79760). Combined with nucleic acid analysis based on agarose-ethidium bromide-stained gels, this sequencing further supports that the dsRNA had been completely eliminated in the virus-cured strain.

10.1128/mBio.01534-20.1 TEXT S1 Identification of the large dsRNA segment of the *M. sympodialis* MsMV1. Note that the designations “TRINITY_DN8321_c1_g1_i1 len = 4613 path=[4591:0-4612] [-1, 4591, -2]” and “TRINITY_DN4986_c1_g1_i1 len = 4601 path=[4579:0-4600] [-1, 4579, -2]” are automatically generated by the software Trinity. We arbitrary used the designations DN8321 and DN4986 to indicate the contigs that represent the *M. sympodialis* MsMV1 mycovirus genome, as identified by BLAST analyses. Considering “TRINITY_DN8321_c1_g1_i1 len = 4613 path=[4591:0-4612] [-1, 4591, -2],” according to the software instructions, TRINITY_DN8321_c1_g1_i1 indicates Trinity read cluster ‘TRINITY_DN8321_c1’, gene ‘g1’, and isoform ‘i1’. ‘Len’ indicates the length of the cluster (contig) in base pairs, and path information indicates the path traversed in the Trinity compacted de Bruijn graph to construct that transcript. Download Text S1, DOCX file, 0.02 MB. Copyright © 2020 Clancey et al. 2020 Clancey et al. This content is distributed under the terms of the Creative Commons Attribution 4.0 International license.

Moreover, PCR analyses with primers designed to specific regions of the large and small dsRNA segments failed to produce amplicons on *M. sympodialis* KS012 genomic DNA, suggesting that the viral sequence is not integrated in the genome but is rather cytoplasmic. This finding is corroborated by *in silico* BLAST searches that failed to detect either large- or small-segment viral sequences in the *de novo* genome assembly of *M. sympodialis* KS012 (described below).

**FIG 2:**
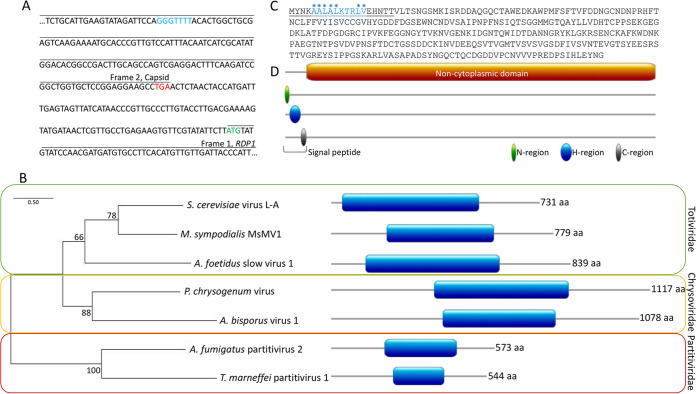
dsRNA segments code for viral protein products. (A) Representative region of the large dsRNA segment illustrating the two open reading frames for the capsid and Rdp1 and the site of the −1 ribosomal frameshift. Stop codon for capsid highlighted in red. Start codon for Rdp1 highlighted in green. Frameshift site highlighted in blue. (B) Phylogenetic analysis of mycovirus Rdp1 sequences. Scale bar represents the number of substitutions per site. Blue boxes on protein diagrams represent the reverse transcriptase (RT) domain of the Rdp1 protein. (C) Protein sequence of the small dsRNA segment. The signal peptide is underlined, and hydrophobic residues are indicated with stars. (D) Pictorial representation of predicted domains of the small dsRNA segment.

Using the ExPASy Translate web tool, the 4.6-kb segment was found to contain two open reading frames (ORFs), distinguished by a −1 ribosomal frameshift, a common feature of totiviruses (Fig. 2A) (33). A BLASTp search of the ORF produced by a frame 1 translation revealed the RNA-dependent RNA polymerase gene (*RDP*) from *Scheffersomyces segobiensis* virus L (SsV L) with 52.69% homology. A BLASTp search of the ORF produced by a frame 2 translation identified the capsid protein from the same *S. segobiensis* virus, in this case with 53.87% homology (34). Phylogenetic analysis of the Rdp1 sequence obtained from RNA-seq grouped it with Rdp1 sequences from other mycoviruses of the *Totiviridae* family (Fig. 2B). Moreover, bidirectional BLAST analysis using the *S. segobiensis* SsV L virus genome as a query against the *M. sympodialis* KS012 transcriptome assembly found the contigs DN8321 and DN4986 (Text S1). These results strongly support that the large dsRNA segment isolated from *M. sympodialis* is a novel mycovirus and encodes proteins required for mycoviral replication and maintenance.

Similarly, the 374-bp query sequence from the smaller dsRNA segment identified via TA cloning was subjected to a BLASTn search against the assembled transcriptomes of the virus-infected and virus-cured *M. sympodialis* strains KS012 and SEC494, respectively. Two highly significant hits (E value 3e−170 and 2e−166) were obtained only against the genome of the virus-infected strain KS012. The two hits are part of two independent contigs (DN7179 of 1,546 bp and DN6052 of 1,479 bp; see Text S2 legend for details), consistent with the size of the small dsRNA segment based on agarose gel electrophoresis. These contigs are identical with the exception of a few nucleotides located at the sequence extremities and outside coding regions (Text S2). Translation of this contig showed a single ORF (Fig. 2C), which when used in a BLASTp search in NCBI returned only weak (≤29% homology, *E* value ≥1.6) hits to the 3′ region of uncharacterized and hypothetical proteins in Sporisorium reilianum and *Streptomyces*, respectively; thus, no significant hits were obtained from NCBI for the small segment.

10.1128/mBio.01534-20.2 TEXT S2 Identification of the small dsRNA segment of the *M. sympodialis* MsMV1. For the contig designations, see explanation in the legend for Text S1. Download Text S2, DOCX file, 0.02 MB. Copyright © 2020 Clancey et al. 2020 Clancey et al. This content is distributed under the terms of the Creative Commons Attribution 4.0 International license.

To infer the function of this virally encoded protein, the predicted amino acid sequence was subjected to domain prediction using InterProScan. A 305-amino-acid noncytoplasmic domain was identified at the C-terminal end of the protein, and a short 19-amino-acid signal peptide is predicted at the N terminus (Fig. 2D). Signal peptides are commonly present at the N terminus of proteins that are destined for secretion. Signal peptides are composed of three regions: an N-region, an H-region, and a C-region. Typically, the N-region contains a stretch of positively charged amino acids, the H-region is the core of the signal peptide that contains a stretch of hydrophobic amino acids, and the C-region contains a stretch of amino acids that are recognized and cleaved by signal peptidases (35). The presence of this signal peptide, and the lack of homology to any protein in the NCBI database, suggests the ∼1.5-kb dsRNA segment from *M. sympodialis* encodes a previously unidentified, novel protein of unknown function that is likely secreted from the cell. Given the data supporting that these dsRNA segments represent a dsRNA virus in *M. sympodialis*, we termed this virus MsMV1 (*Malassezia sympodialis* mycovirus 1).

### MsMV1 infection causes an upregulation of a large number of ribosomal genes.

Total RNA from congenic virus-infected and virus-cured strain pairs KS012 and SEC494 was subjected to analysis of differentially expressed genes in the virus-infected strain compared to its cured counterpart. For RNA-seq analysis, three biological replicates of each sample were analyzed. RNA-seq reads of virus-infected and virus-cured strains KS012 and SEC494 were mapped against the reference *M. sympodialis* genome ATCC 42132 using HiSat2. Transcript abundance was estimated through StringTie, and differentially expressed genes with a false-discovery rate (FDR) <0.05 and a log_2_ fold change ±1 were identified with EdgeR (36, 37).

**FIG 3:**
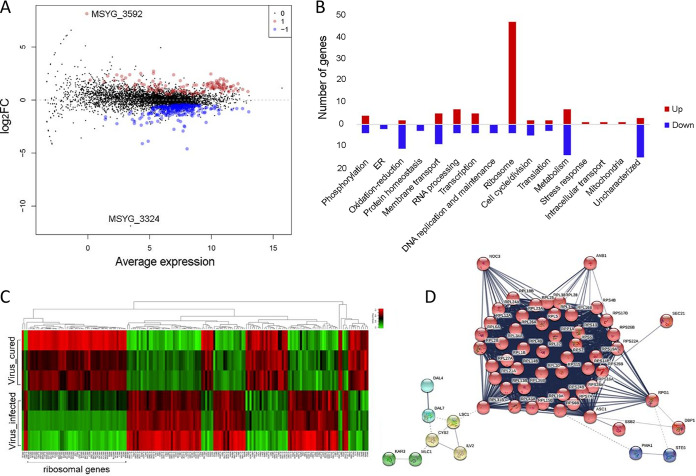
RNA-seq reveals transcriptional rewiring in virus-infected *M. sympodialis* strain. (A) Dot plot of log_2_ fold change in gene expression in the virus-infected strain KS012. Red and blue dots represent upregulated and downregulated genes that pass a false-discovery rate (FDR) of 5%, respectively. Gray dots indicate genes that do not meet the FDR. The virus-cured strain SEC494 was used as the baseline for gene expression, and a gene is marked as differentially expressed in the virus-infected strain KS012 if expression differs from SEC494. (B) Number of genes upregulated (red) and downregulated (blue) categorized by gene ontology. (C) Heatmap of the three RNA-seq technical replicates of virus-infected and virus-cured strains. Green represents upregulated genes, and red represents downregulated genes. (D) Gene network map of upregulated genes in virus-infected strain. Genes are clustered by color based on the Markov-Cluster (MCL) inflation parameter.

The virus-infected strain displayed differential expression of 172 genes in comparison to the virus-cured strain, with 89 genes being upregulated (log_2_ fold change between 1 and 8.14) and 83 genes being downregulated (log_2_ fold change between −1 and −4.6) (Fig. 3A). Among the proteins differentially upregulated, ∼50% are involved in ribosomal biogenesis and translation (Fig. 3B). The remaining genes are involved in DNA replication and maintenance, transcription, stress responses, and other cellular metabolic processes. The most highly upregulated gene in the virus-infected strain, with a log_2_ fold change of 8.14, was *MSYG_3592*, which encodes an uncharacterized mediator complex protein. The mediator complex is a transcriptional coactivator that interacts with transcription factors and RNA polymerase II, which transcribes all protein-coding and most noncoding RNA genes (38). The change in expression of this gene, combined with the upregulation of phosphorylation activity, ribosomal biogenesis, and cytoplasmic translation, indicates a large transcriptional shift in the virus-infected strain. Downregulated genes included those involved in metabolic processes, oxidation-reduction processes, transmembrane transport, cell cycle, and cell division and many uncharacterized transcripts. In particular, the most downregulated gene that passed the FDR <0.05 threshold with a log_2_ fold change of −4.6 is an endoplasmic reticulum (ER) membrane protein, *MSYG_1988*, a gene whose predicted product is involved in the movement of substances across the ER membrane and/or protein synthesis. The second most downregulated gene is *MSYG_0220* (log_2_ fold change −4.1), which is an ortholog of S. cerevisiae *CDC39.* Cdc39 is a component of the Ccr4-NotI complex that, in addition to its role as a global transcriptional regulator, regulates mRNA decay. These data suggest that elimination of aberrant mRNA transcripts may be inhibited in the virus-infected strain. Interestingly, we identified a gene (*MSYG_3324*) with the lowest log_2_ fold change (−11.88) that did not pass the statistically significant threshold of FDR <0.05. The gene *MSYG_3324* encodes a hypothetical protein that has a guanine nucleotide exchange factor domain, leucine-rich repeats of the RNase inhibitor (RI)-like subfamily, and an N-terminal herpes BLLF1 superfamily domain, which consists of the BLLF1 viral late glycoprotein that is the most abundantly expressed glycoprotein in the viral envelope of the herpesviruses. Strikingly, BLLF1 represents a major antigen responsible for production of neutralizing viral antibodies *in vivo* (39), and we speculate that it might be strongly downregulated by the MsMV1 mycovirus as a mechanism to dampen *M. sympodialis* antiviral defense systems. Overall, our RNA-seq data set is clearly indicative of a significant transcriptional rewiring of *M. sympodialis* in the presence of the dsRNA mycovirus MsMV1.

The three technical replicates of each strain produced highly similar expression profiles, which are distinct from one another (Fig. 3C). The largest cluster of upregulated genes in the virus-infected strains included ribosomal components, and a gene network map shows the tight interaction of these ribosomal components (Fig. 3D). The large, central cluster consists of ribosomal proteins that are regulated by translation initiation factors Anb1 and Rpg1, and also Noc3, which binds ribosomal precursors to mediate their maturation and intranuclear transport.

One question that we sought to answer was whether the conditions employed to cure the virus changed the strain in any way other than virus elimination. We generated whole-genome Illumina data and then performed genome-wide analyses and single nucleotide polymorphism (SNP) calling of the *M. sympodialis* cured strain SEC494, comparing it to the virus-infected strain KS012. Only two nucleotide changes unique to the virus-cured isolate SEC494 were identified. These include mutations in the coding regions of *KAE1*, an ATPase, and *CRM1*, a karyopherin responsible for the transport of molecules between the cytoplasm and the nucleus (Table S1 and Text S3). As these mutations were found only in the virus-cured strain, they may be a consequence of the “curing treatment,” and we predict their impact on fitness to be minimal given similar growth characteristics of the two strains (Table S1; Text S3). Moreover, the assembled KS012 and SEC494 genomes are colinear with the reference *M. sympodialis* genome ATCC 42132 (Fig. S2A to C), and no change in ploidy or aneuploidy was observed for any chromosome (Fig. S2D).

10.1128/mBio.01534-20.3 TEXT S3 Screenshot of the variants presents in the coding regions of the genes *KAE1* and *CRM1* in the *M. sympodialis* strain SEC494. Download Text S3, DOCX file, 0.3 MB. Copyright © 2020 Clancey et al. 2020 Clancey et al. This content is distributed under the terms of the Creative Commons Attribution 4.0 International license.

10.1128/mBio.01534-20.5 FIG S2 Genomic comparisons of *M. sympodialis* virus-infected and virus-cured strains compared to the reference strain ATCC 42132. (A to C) Dot plot comparisons of the *M. sympodialis* strain ATCC 42132 with the virus-infected *M. sympodialis* KS012 (A) and the virus-cured SEC494 (B), and of the *M. sympodialis* isogenic strains KS012 and SEC494. (C) Read coverage of the *M. sympodialis* strains KS012 and virus-cured SEC494 compared to the haploid reference strain ATCC 42132. Download FIG S2, TIF file, 0.6 MB. Copyright © 2020 Clancey et al. 2020 Clancey et al. This content is distributed under the terms of the Creative Commons Attribution 4.0 International license.

10.1128/mBio.01534-20.8 TABLE S1 Variants present only in the *M. sympodialis* virus-cured SEC494 compared to the virus-infected KS012. Download Table S1, XLSX file, 0.01 MB. Copyright © 2020 Clancey et al. 2020 Clancey et al. This content is distributed under the terms of the Creative Commons Attribution 4.0 International license.

### *Malassezia* mycovirus does not impact pathogenicity in an epicutaneous murine model.

In a recent study, Sparber and colleagues described a murine epicutaneous infection model that involves application of *Malassezia* to the mouse dorsal ear following mild tape stripping (40). Colonization of host tissue (fungal load) is determined by plating homogenized tissue onto agar plates and counting CFU; an increase in ear thickness is used as a measurement for skin inflammation. When mice were challenged with congenic virus-infected and virus-cured strains in this model, only the virus-infected strain KS012 displayed increased colonization compared to its virus-cured counterpart, while there was no difference in the other strain pairs tested by day 4 postinfection (Fig. S3A). No change in ear thickening (as a readout for inflammation) was observed in response to the virus-infected strains compared to their cured counterparts. To control for the temperature exposure in the virus-cured strains, a strain without MsMV1 was passaged at 37°C. No changes in skin colonization or ear thickening were observed in the heat-treated virus-uninfected counterpart when tested in the epicutaneous infection model (Fig. S3A). To determine if there was a difference in the immune responses elicited by the virus-infected and virus-cured strains, we assessed the expression of interleukin-17 (IL-17), a key mediator of the host response against *Malassezia* on the skin (40). However, we could not detect any statistically significant differences in cytokine induction in response to virus-infected and virus-cured strains (Fig. S3C), indicating that IL-17 expression is not regulated by the virus and not responsible for the observed differences in fungal load observed for strain KS012. We also assessed expression of IFN-β, a central player in antiviral immunity but could not measure expression above the basal levels detected in the skin of naive control animals (Fig. S3C). The same was also true at earlier or later time points after infection (Fig. S3D and E). Similarly, the heat-untreated and heat-treated virus-uninfected control strain did not display differential IL-17 or IFN-β expression (Fig. S3C).

10.1128/mBio.01534-20.6 FIG S3 Select virus-infected strains exhibit increased ability to colonize skin in a murine ear skin infection model. (A) Fungal load, (B) ear thickening, and (C) cytokine expression in the skin of mice that were epicutaneously infected with the indicated virus-infected and virus-cured strain pairs for 4 days, (D) 2 days, or (E) 7 days. The mean + SEM for each group are indicated. Data in panels A to C are pooled from two independent experiments with *n* = 6 mice for all groups, except *n* = 3 for KS015 and SEC497. DL, detection limit. Statistics were calculated using one-way analysis of variance (ANOVA). *, *P < *0.05; ****, *P < *0.0001. Black bars below the *x* axis labels group virus-infected/virus-cured strain pairs. “+” indicates virus infected; “−” indicates virus cured. Download FIG S3, TIF file, 0.4 MB. Copyright © 2020 Clancey et al. 2020 Clancey et al. This content is distributed under the terms of the Creative Commons Attribution 4.0 International license.

### Virus-infected strains induce beta interferon expression in BMDMs in a TLR3-independent manner.

**FIG 4:**
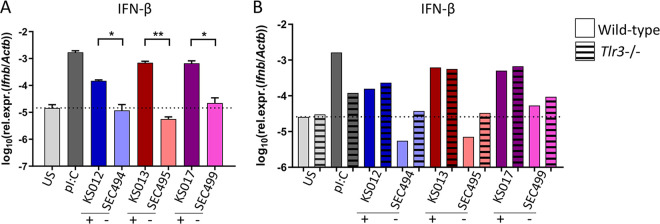
MsMV1 dsRNA virus induces beta interferon (IFN-β) expression in cultured macrophages in a TLR3-independent manner. (A) IFN-β expression in bone marrow-derived macrophages (BMDMs) after infection with the indicated virus-infected and virus-cured strains at an MOI of 5 for 24 h. Bars are the mean + SEM for four replicate samples with each being the pool of two separate wells. Data are pooled from two independent experiments. (B) IFN-β expression by wild-type (solid bars) and *Tlr3^−/−^* (striped bars) BMDMs after infection with the indicated virus-infected and virus-cured strains at an MOI of 5 for 24 h. Bars are a pool of two wells. Data are representative of one out of two independent experiments. Poly(I:C) was included as a positive control; US, unstimulated. The dotted line indicates the expression levels under unstimulated conditions. Statistics were calculated using unpaired Student’s *t* test. *, *P* < 0.05; **, *P* < 0.01. Black bars below the *x* axis labels group virus-infected/virus-cured strain pairs. “+” indicates virus infected; “−” indicates virus cured.

Given its close relationship with the skin, *Malassezia* primarily interacts with keratinocytes and tissue-resident mononuclear phagocytes, including macrophages. Therefore, we assessed the interaction of *M. sympodialis* virus-infected and virus-cured strains with macrophages. Bone marrow-derived macrophages (BMDMs) are mature, differentiated macrophage that are suitable for host-pathogen interaction studies (41). Beta interferon (IFN-β) expression in BMDMs was measured by reverse transcriptase quantitative PCR (RT-qPCR) following incubation with virus-infected and virus-cured *M. sympodialis* strains for 24 h. The presence of MsMV1 induced significantly more IFN-β expression in BMDMs than did virus-cured strains (Fig. 4A).

In leishmaniasis, a parasitic disease caused by *Leishmania*, an elevated burden of *Leishmania* RNA virus-1 (LRV1) in the parasite is associated with parasite dissemination and increased disease severity (42, 43). Toll-like receptors (TLRs) are innate immune-recognition receptors known to play a role in viral infection, and TLR3, which recognizes and responds to dsRNA (44), was found to be required for the induction of proinflammatory factors and disease exacerbation in leishmaniasis (42). Given the relationship between TLR3 and dsRNA recognition, TLR3-deficient BMDMs were incubated with the virus-infected and virus-cured *Malassezia* strains. In contrast to observations with LRV1, the increased production of IFN-β by MsMV1-infected strains was not dependent upon TLR3, as deletion of this gene in BMDMs did not result in a loss of IFN-β expression by the virus-infected strains (Fig. 4B).

BMDM viability and macrophage activation, as determined by CD86^+^ surface expression and TNF expression, was also assessed (Fig. S4A). No significant differences were found in BMDMs incubated with virus-infected and virus-cured *M. sympodialis* strains, and deletion of TLR3 did not change this result (Fig. S4C). The seemingly enhanced induction of CD86 by the virus-cured strain SEC494 (Fig. S4A) was not reproducible in independent repeat experiments, not paralleled by other readouts of macrophage activation, and not consistent with the results obtained with other virus-infected and virus-cured strain pairs. Therefore, it seems unlikely to be related to the absence of the virus.

10.1128/mBio.01534-20.7 FIG S4 Macrophage viability, CD86 expression, and TNF expression are not affected by the presence of MsMV1 in *M. sympodialis* strains. (A) BMDMs were infected with the indicated virus-infected and virus-cured strains at an MOI of 1 and analyzed for viability and CD86 surface expression by flow cytometry at 16 h postinfection or infected at an MOI of 5 and analyzed for TNF expression by RT-qPCR at 24 h postinfection. Each bar represents the mean + SEM for four samples, each being the pool of two separate wells. Data are pooled from two independent experiments. The dotted line indicates the expression levels under unstimulated conditions. (B) Flow cytometry gating strategy used to assess BMDM viability (top row) and CD86 expression (bottom row). (C) BMDM viability, CD86 surface expression, and TNF expression of wild-type and *Tlr3^−/−^* BMDMs infected with the indicated virus-infected and virus-cured strains and analyzed as in panel A. Data are from one out of two representative experiments with each value being the pool of two wells. Statistics were calculated using an unpaired Student *t* test. *, *P* < 0.05. “+” superscripts indicate virus-infected strains; “−” superscripts indicate virus cured. Download FIG S4, TIF file, 0.7 MB. Copyright © 2020 Clancey et al. 2020 Clancey et al. This content is distributed under the terms of the Creative Commons Attribution 4.0 International license.

## DISCUSSION

Mycoviruses are proving to be much more ubiquitous in nature than previously appreciated. Fungi harboring mycoviruses display a range of phenotypes, which in some cases can prove beneficial or detrimental for the host plant or animal. For instance, *C. parasitica* carries the CHV1 virus that causes reduced growth and pathogenicity of the fungus. Therefore, trees infected with virus-infected strains of *C. parasitica* display smaller cankers than trees infected with virus-free strains (16). Application of hypovirus-infected strains to cankers has proven to be an effective biocontrol agent as it halted the decimation of the chestnut tree in North America (17). Conversely, virus-infected Leishmania guyanensis exacerbates leishmaniasis in mice and increases parasitemia, leading to the dissemination and metastasis of the parasite from the infection site (42).

Here, we describe the discovery of dsRNA mycoviruses in five *Malassezia* species, *M. sympodialis*, *M. pachydermatis*, *M. globosa*, *M. obtusa*, and *M. yamatoensis* (Fig. 1A and B and see Fig. S1 in the supplemental material). RNA molecules were isolated in 1994 from a collection of *M. pachydermatis* isolates (69), and although they might be the first evidence of mycoviruses in *Malassezia*, their characterization was not carried out. In the present study we characterize in detail the MsMV1 virus from *M. sympodialis.* RNA sequencing revealed that the large dsRNA segment from *M. sympodialis* is of viral origin and contains two open reading frames that encode the Rdp1 and capsid proteins, which are translated by a −1 ribosomal frameshift, a hallmark feature of the *Totiviridae* virus family (Fig. 2A) (33). Accordingly, phylogenetic analysis of the Rdp1 open reading frame groups it most closely with other mycoviruses from the *Totiviridae* family (Fig. 2B). Using the small dsRNA segment sequence as a query, no significant matches were found in the NCBI database, suggesting the satellite virus is unique to this fungus and could serve a specialized role. Taken together, these data support the discovery of a novel mycovirus in the important skin fungus *M. sympodialis*. Further investigation will be required to characterize the presumptive mycoviruses in the other *Malassezia* species.

We find that exposure to high temperature (37°C) or to an antimicrobial agent (biphenyl) cures *M. sympodialis* of MsMV1 (Fig. 1C). These are two conditions frequently encountered by fungi. Systemic human patient fungal isolates are exposed to high temperature during infection, and isolates from both patients and the environment are often purified on media containing antimicrobials to inhibit bacterial or filamentous fungal growth from the host or environment. It is possible these typical microbiological isolation methods may be inducing mycovirus curing during isolation. Taken together with the growing reports on the discovery of novel mycoviruses, mycoviruses may be much more abundant than currently appreciated. Not all of the *Malassezia* isolates analyzed in this study harbored a mycovirus, and this could reflect either natural diversity or curing during isolation. It is not clear why these two conditions cause virus curing. In the case of high temperature, this stressful condition may alter cellular physiology, rendering the cell a less favorable host for the mycovirus. Viral replication and maintenance, while neutral at 30°C, could interfere with cellular processes required for survival at 37°C. Moreover, high temperature is known to increase heat shock and misfolded proteins in the cell (45), and if full Hsp90 function is required for viral replication, then the virus could be lost due to the overburdened Hsp90.

RNA sequencing of virus-infected and virus-cured strains revealed major differences in the transcriptional profile between the two strains. Most notable is the upregulation of a large number of ribosomal components in the virus-infected strains, suggesting an increased translational activity induced by the mycovirus. A similar result was found with the plant fungus Sclerotinia sclerotiorum, which harbors the SsHV2-L hypovirus, where rRNA metabolic processes and cellular component biogenesis were the classes of the most upregulated genes in virus-infected strains (46). Alternatively, the opposite has been observed in S. cerevisiae, where virus infection is associated with the downregulation of ribosome biogenesis and stress response, suggesting that in S. cerevisiae, virus-infected cells may be less stressed due to host protection systems for the killer virus (47).

Genes involved in oxidation-reduction processes are another category that was downregulated in the virus-infected strain. Denitrification, the biological process of converting nitrate to ammonia through reduction, which is then broken down into nitrogen, nitrous oxide, and nitric oxide (NO), is an important pathway in living organisms. NO is an important signaling molecule in bacteria and fungi. Furthermore, NO production is positively correlated with aflatoxin production in Aspergillus nidulans, where deletion of flavohemoglobin, a nitric oxide reductase, leads to reduced sterigmatocystin production and *aflR* expression (48). Flavohemoglobin, encoded by the *YHB1* gene in *M. sympodialis*, does not appear to play a role in putative toxin-producing strains as it is downregulated only 0.69-fold in MsMV1-infected strains. Recent work from Wisecaver and colleagues as well as our lab has identified that *Malassezia* acquired flavohemoglobin via a horizontal gene transfer event from *Actinobacteria* (48, 49). A likely explanation for the difference in the role of flavohemoglobin between A. nidulans and *Malassezia* is that the gene was not endogenous to *Malassezia* and rather was acquired from an exogenous bacterial source, and therefore may not have been adapted to play a regulatory role with the virus. The presence of the virus may be inducing the upregulation of transcriptional and translational machinery in order to synthesize components required for viral replication, maintenance, and persistence. Analysis of gene expression in additional virus-infected strains as well as virus-uninfected strains would further confirm the gene expression changes imparted by the virus. How this signal is translated from the virus to the fungus is an interesting area for future research.

RNAi is a common antiviral defense system that operates in fungi to combat viral persistence in the cell. As expected, *C. parasitica* CHV1-infected cells exhibit an upregulation of silencing-related genes in an effort to reduce the viral burden or clear the virus. Alternatively, a different phenomenon occurs in *T. marneffei* TmPV1-infected cells where the virus can subsequently dampen expression of silencing-related genes and blunt the cellular RNAi defense mechanism (4, 50, 51). *M. sympodialis* has lost the canonical components of RNAi, and we hypothesize that the loss of RNAi may be contributing to mycovirus replication and persistence. Furthermore, we observed changes in genes related to regulating mRNA processing. One significantly downregulated gene is involved in mRNA decay, *MSYG_0220* (*CDC39*). As described earlier, during the virus replication cycle, a ssRNA intermediate is formed prior to virion assembly (13) and is vulnerable to fungal defense mechanisms. Suppressing the expression of genes involved in eliminating anomalous mRNA transcripts could be a strategy for the virus to survive in the fungal cell. Interestingly, the RNA-seq analysis identified the downregulation of *MSYG_03961* (*CPR3*) in virus-infected *M. sympodialis* cells. *CPR3* is a gene that encodes a mitochondrial cyclophilin (52) that could be linked to pathogenesis via established roles of mitochondria in basidiomycete pathogens (53). Although the passing at high temperature did not induce many genomic changes, it is possible that epigenetic changes occurred during the treatment that resulted in changes in gene expression, and further studies are needed to address this question.

Mycoviruses impact fungal virulence in different ways. Mycoviruses can be asymptomatic, whereas others induce either a hypo- or hypervirulent state in the host fungus. We employed a recently developed murine model for *Malassezia* in which the fungus is applied onto the mouse ear following mild barrier disruption to replicate hallmarks of atopic dermatitis (40). We found one of two virus-infected strains that displayed an enhanced ability to colonize the murine skin, compared to its virus-cured counterpart, but did not observe any differences in inflammation or cytokine production (Fig. S3A, B, and C). Promisingly, cultured macrophages responded differently to virus-infected and virus-cured strains in which more IFN-β was produced from BMDMs incubated with virus-infected strains than from those incubated with virus-cured strains (Fig. 4A). Other parameters such as BMDM viability and BMDM activation were measured in parallel, and no difference was detected between virus-infected and virus-cured strains (Fig. S4A). We conclude that under certain circumstances, MsMV1 appears to provide an enhanced ability to colonize skin, and increased IFN-β in cultured macrophages is a common characteristic of all virus-infected strains tested, thus suggesting some role in the pathogenicity of *M. sympodialis.* However, the degree to which the virus plays a role is still uncertain. It is possible that characteristics in the genomic background of the *M. sympodialis* virus-infected strain influence the degree to which the virus exacerbates disease. Perhaps there are features of the KS013 genome that allow it to better control viral load of MsMV1, and so it does not impart the same growth advantage on the skin. As KS012 was the only virus-infected genome sequenced in this study, we do not know if there are any differences between it and KS013 that would account for the differences observed in skin colonization experiments.

Due to the role of IFN-β in the stimulation of antiviral and antiproliferative products (54), it is possible that the production of IFN-β may lead to enhanced clearing of virus-infected fungal cells from macrophages. TLR3 binds dsRNA in mammalian cells and was shown to play a critical role in disease exacerbation in leishmaniasis when the infecting *Leishmania* strain harbors a mycovirus (42, 43). In the case of *M. sympodialis*, we did not observe a requirement of TLR3 for IFN-β production in response to virus-infected strains (Fig. 4B). We posit that dsRNA recognition by *Malassezia* may occur through an alternative virus recognition pathway yet to be elucidated that may recognize mycoviral dsRNA as a fungal pathogen-associated molecular pattern (PAMP).

In conclusion, we report for the first time evidence of mycoviruses infecting the fungal genus *Malassezia*. Although the target of the putative satellite toxin is unknown, it induces an immune response in macrophages. Coincubation of the virus-infected *M. sympodialis* with virus-cured *M. sympodialis* and bacterial species (Escherichia coli, *Corynebacterium* spp., and *Staphylococcus* spp.) failed to reveal antifungal or antibacterial properties of the virus-infected *M. sympodialis* strains. Determining if changes in the expression of metabolic genes in the virus-infected strains result in resistance or susceptibility to certain environmental conditions would be interesting to investigate in the future. More research will be required to determine if this possible mycovirus toxin plays a role in common skin disorders caused by *Malassezia* by targeting the host, bacteria, or fungi of the skin microbiome.

## MATERIALS AND METHODS

### Strains.

*Malassezia sympodialis* strains were stored at −80°C in 25% glycerol stocks and maintained on modified Dixon’s (mDixon) medium at 30°C. mDixon medium is composed of 3.6% malt extract, 1% mycological peptone, 1% desiccated ox bile, 1% Tween 60, 0.4% glycerol, and 2% agar for solid medium. Strains used in dsRNA gel images are listed in Table 1, and other strains used in this study are listed in Table 2.

**TABLE 2:** Strains used in this study

Strain name	Details
KS012	*M. sympodialis*; MsMV1 infected
KS013	*M. sympodialis*; MsMV1 infected
KS016	*M. sympodialis*; MsMV1 infected
KS017	*M. sympodialis*; MsMV1 infected
SEC494	*M. sympodialis*; MsMV1 temperature-cured congenic strain of KS012
SEC495	*M. sympodialis*; MsMV1 temperature-cured congenic strain of KS013
SEC498	*M. sympodialis*; MsMV1 temperature-cured congenic strain of KS016
SEC499	*M. sympodialis*; MsMV1 temperature-cured congenic strain of KS017
SEC554	*M. sympodialis*; MsMV1 biphenyl-passaged congenic strain of KS012
SEC555	*M. sympodialis*; MsMV1 biphenyl-cured congenic strain of KS013
SEC557	*M. sympodialis*; MsMV1 biphenyl-cured congenic strain of KS016
SEC558	*M. sympodialis*; MsMV1 biphenyl-passaged congenic strain of KS017

### RNA extraction and dsRNA enrichment.

Total RNA was extracted from *M. sympodialis* liquid cultures as follows. Five-milliliter liquid mDixon cultures were grown at 30°C until saturation, approximately 3 to 4 days. Cultures were pelleted at 3,000 rpm in a tabletop centrifuge for 3 min and washed with 5 ml distilled water (dH_2_O), and pellets were frozen at −80°C until lyophilization overnight. Following lyophilization, pellets were broken up with 3-mm glass beads and the RNA extraction was performed using TRIzol (ThermoFisher, catalog no. 15596026) according to the manufacturer’s instructions. RNA was resuspended in 50 μl diethyl pyrocarbonate (DEPC) water. RNA was treated with Turbo DNase (ThermoFisher, catalog no. AM2238) according to manufacturer’s instructions and resuspended in 50 μl DEPC water. ssRNA precipitation/dsRNA enrichment was done overnight at −20°C by combining 25 μl of DNase-treated RNA, with 28 μl 8 M LiCl ([final] = 2.8 M) and 27 μl DEPC water for a final volume of 80 μl. The mixture was centrifuged at 4°C for 10 min, and the supernatant was recovered and transferred to a fresh Eppendorf tube (the pellet can be saved and resuspended in 25 μl DEPC water as an ssRNA control). To the supernatant, 7.5 μl 3 M NaCl ([final] = 0.1 M) and 187.5 μl 100% ethanol ([final] = 85%) were added and placed at −20°C for at least 1 h. dsRNA was pelleted at 4°C for 15 min and resuspended in 25 μl DEPC water. To visualize dsRNA and ssRNA bands, 10 μl was electrophoresed on an 0.8% agarose gel stained with 5 μg/ml of ethidium bromide.

### TA cloning of dsRNA segments.

Large and small dsRNA segments were purified from *M. sympodialis* strains KS012, KS013, KS016, and KS017 as described above. Large and small dsRNA segments were excised from the agarose gel and purified via gel extraction using the QIAquick gel extraction kit (Qiagen, catalog no. 28704). 3′ adapter sequences from the Illumina TruSeq small RNA library preparation kit (Illumina, catalog no. RS-200) were ligated onto the dsRNA species by mixing together 1 μl of adapter with 1 μg dsRNA in a 6-μl reaction mixture and heated at 70°C for 2 min. Following heating, tubes were immediately placed on ice. To the adapter reaction mixture, 2 μl of HML ligation buffer, 1 μl RNasin, and 1 μl T4 RNA ligase 2 (truncated) were added and mixed by pipetting. The solution was incubated for 1 h at 28°C.

cDNA synthesis was achieved by combining 5 μl of adapter-ligated dsRNA with 1 μl RT primer and heated at 70°C for 2 min and then immediately placed on ice. Next, the adapter-ligated/RT primer mixture was used as the template with the AffinityScript cDNA synthesis kit (Agilent, catalog no. 600559) with the following reaction conditions: 25°C for 5 min, 55°C for 15 min, 95°C for 5 min. cDNA was PCR amplified using the RT primer, cloned into the pCR-Blunt vector using the Zero Blunt PCR cloning kit (Invitrogen, catalog no. K270020), and transformed into E. coli with One Shot TOP10 chemically competent E. coli (Invitrogen, catalog no. C404003). Plasmids were extracted from purified bacterial colonies and subjected to Sanger sequencing (Genewiz) using universal M13F and M13R primers.

### RNA sequencing.

Five-milliliter liquid mDixon cultures were grown at 30°C for 3 days. Cells were pelleted at 3,000 rpm in a tabletop centrifuge for 3 min, washed with 5 ml dH_2_O, and resuspended in 1 ml dH_2_O. The 1-ml cell suspension was transferred to a bead beating tube with acid-washed 425- to 600-μm beads. Cells were pelleted with beads in a microcentrifuge at 6,000 rpm for 2 min. The supernatant was removed, and the cell pellet was flash frozen in liquid nitrogen. Frozen bead/cell pellets were stored at −80°C until RNA extraction. To prepare RNA, bead/cell pellets were thawed on ice and 1 ml TRIzol (ThermoFisher, catalog no. 15596026) was added. Cells were subjected to bead beating 10 times in 1-min “on,” 1-min “rest” cycles. Following bead beating, 200 μl of chloroform was added and the RNA extraction continued as described in the manufacturer’s instructions. Final RNA pellets were treated with Turbo DNase (ThermoFisher, catalog no. AM2238) according to manufacturer’s instructions, and RNA was resuspended in 50 μl of nuclease-free water (Promega, catalog no. P119A). Illumina 150-bp paired-end (PE) libraries were prepared using the TruSeq stranded total RNA-seq kit in combination with the Illumina RiboZero yeast kit. Library preparation and RNA sequencing were performed at the Duke University Center for Genomic and Computation Biology.

After sequencing, Illumina raw reads were trimmed with Trimmomatic to remove Illumina adapters (55). For the identification of the dsRNA mycovirus genome, one sequencing replicate of both the virus-infected and virus-cured strains was assembled into a transcriptome using Trinity (56), which was run on Galaxy (57). The generated transcriptome was searched using the TA-cloned sequences derived from both the large and small dsRNA segments. *In silico* functional characterization of the mycovirus sequences was performed through BLAST analyses and InterProScan (https://www.ebi.ac.uk/interpro/search/sequence/). For RNA-seq analysis, the most recent *M. sympodialis* strain ATCC 42132 genome assembly and annotation were used as a reference (30). Illumina reads from three independent biological replicates were trimmed with Trimmomatic as reported above, and cleaned reads were mapped to the *M. sympodialis* reference genome using HiSat. Generated .bam files were used to run StringTie with the *M. sympodialis* annotation as guide and the –e option to provide the correct output for the statistical analysis (36). Read count information for statistical analysis was extracted using a provided Python script (prepDE.py). EdgeR was used to determine the differentially expressed genes (DEGs) in the *M. sympodialis* virus-infected strain KS012 compared to its cured counterpart SEC494. StringTie and EdgeR were run on Galaxy (57). DEGs were considered those with na FDR <0.05 and with a log_2_ fold change ±1. Functional annotation of the DEGs was performed using the Blast2GO pipeline, which includes the BLASTx against the NCBI nonredundant protein database, gene ontology (GO) annotation, and InterProScan (58). The heatmap for the DEGs was generated using the web-based server heatmapper with the average linkage for hierarchical clustering (http://www.heatmapper.ca/expression/), and gene network interaction were determined using STRING (https://string-db.org/cgi/input.pl) with the S. cerevisiae protein set as reference.

### Whole-genome DNA sequencing and assembly.

Twenty-five-milliliter liquid mDixon cultures of *M. sympodialis* KS012 and SEC494 were grown at 30°C for 3 days. Cells were pelleted at 3,000 rpm in a tabletop centrifuge for 3 min and washed with 10 ml dH_2_O, and cell pellets were frozen at −80°C. Cell pellets were lyophilized overnight, pulverized using 3-mm glass beads, and subjected to genomic DNA extraction using the CTAB method (59). Library preparation and 50-bp PE Illumina sequencing were performed at the Duke University Center for Genomic and Computation Biology. Illumina reads for *M. sympodialis* KS012 and SEC494 were assembled using SPAdes with default parameters (60). Dot plot comparisons were carried out using nucmer with the –nosymplify, –filter, and –fat parameters; nucmer was run on Galaxy. The *de novo* genome assembly of *M. sympodialis* KS012 was used for BLAST searches using both large and small dsRNA viral sequences.

Illumina reads of the *M. sympodialis* virus-infected strain KS012 and virus-cured SEC494 were mapped to the *M. sympodialis* reference ATCC 42132 using BWA (61), and the resulting .sam file was converted to .bam with SAMtools (62). Bam files were converted in .tdf format using IGV, and the read coverage for *M. sympodialis* virus-infected strain KS012 and virus-cured SEC494 was visualized in the IGV browser using the *M. sympodialis* ATCC 42132 strain as reference. For variant analysis, the .bam files were generated as described above, and the *M. sympodialis* reference ATCC 42132 genome was used to run SAMtools mpileup; variant analysis was carried out using the bcftools call option; the resulting .vcf file was merged with the *M. sympodialis* annotation using the variant effector predictor software present on the EnsemblFungi database (https://fungi.ensembl.org/Malassezia_sympodialis_atcc_42132_gca_900149145/Info/Index).

### Phylogenetic analysis.

The MsMV1 Rdp1 was aligned with representative viral Rdp1 sequences found on NCBI using the Mega7 software. Phylogenies were built using 100 bootstrap values using the maximum likelihood method based on the Tamura-Nei model (63). Protein domains were predicted using NCBI BLASTp, and images were created using the MyDomain Image Creator from PROSITE on the ExPASy Bioinformatics resource portal.

### Murine topical infection model.

All mouse experiments in this study were conducted in strict accordance with the guidelines of the Swiss Animals Protection Law and were performed under the protocols approved by the Veterinary office of the Canton of Zurich, Switzerland (license number ZH168/2018). All efforts were made to minimize suffering and ensure the highest ethical and humane standards according to the 3R principles. Wild-type (WT) C57BL/6J mice were purchased by Janvier Elevage and maintained at the Laboratory Animal Science Center of the University of Zurich, Zurich, Switzerland. Mice were used at 6 to 12 weeks in sex- and age-matched groups. Epicutaneous infection of the mouse ear skin was performed as described previously (40, 64). Briefly, *Malassezia* strains were grown for 3 to 4 days at 30°C, 180 rpm, in liquid mDixon medium. Cells were washed in phosphate-buffered saline (PBS) and suspended in native olive oil at a density of 20 OD_A600_ (optical density at 600 nm)/ml. A 100-μl suspension (corresponding to 2 OD_A600_) of yeast cells was applied topically onto the dorsal ear skin that was previously disrupted by mild tape stripping while mice were anaesthetized. Ear thickness was monitored before and after infection using an Oditest S0247 0- to 5-mm measurement device (Kroeplin), and the increase was calculated by subtracting the values measured prior to the experiments from those measured at the endpoint. For each animal, the mean of the ear thickness from both ears was calculated. For determining the fungal loads in the skin, tissue was transferred in water supplemented with 0.05% Nonidet P-40 (AxonLab), homogenized and plated on modified Dixon agar, and incubated at 30°C for 3 to 4 days.

### Macrophage infection *in vitro*.

Macrophages were generated from WT C57BL/6J and *Tlr3^−/−^* (65) (Swiss Immunological Mouse Repository [SwImMR] hosted by the University of Zurich) bone marrow cells in 20% L929 conditioned medium and 80% RPMI 1640 medium supplemented with glutamine, penicillin, streptomycin, 2-mercaptoethanol, sodium pyruvate, nonessential amino acids, HEPES, and 10% (vol/vol) heat-inactivated fetal calf serum (FCS). After 6 days of culture, cells were detached using PBS-5% FCS-2.5 mM EDTA and seeded at a density of 10^5^ cells/100 μl in 96-well tissue culture plates (for transcript analysis) and 10^6^ cells/1 ml in 24-well plates (for fluorescence-activated cell sorting [FACS] analysis). Cells were rested overnight and infected the next day with *Malassezia* at the indicated multiplicity of infection (MOI) for 16 h (for FACS analysis) or for 24 h (for transcript analysis). Poly(I:C) (50 μg/ml) was included as a control.

### Flow cytometry.

Macrophages were gently detached by using cell scrapers and stained with antibodies directed against CD11b (clone M1/70), F4/80 (clone BM8), CD86 (clone GL-1), and major histocompatibility complex class II (MHC-II) (clone M5/114.15.2) in PBS supplemented with 1% FCS, 5 mM EDTA, and 0.02% NaN_3_. LIVE/DEAD Near IR stain (Life Technologies) was used for exclusion of dead cells. Cells were acquired on a FACS Cytoflex (Beckman Coulter), and the data were analyzed with FlowJo software (FlowJo LLC). The gating of the flow cytometric data was performed according to the “Guidelines for the use of flow cytometry and cell sorting in immunological studies” (66), including pregating on viable and single cells for analysis.

### RNA extraction and quantitative RT-PCR.

Isolation of total RNA from ear skin and from BMDM cultures was carried out according to standard protocols using TRI reagent (Sigma-Aldrich). cDNA was generated by RevertAid reverse transcriptase (ThermoFisher). Quantitative PCR was performed using SYBR green (Roche) and a QuantStudio 7 Flex (Life Technologies) instrument. The primers used for qPCR were as follows: *Il17a*, forward 5′-GCTCCAGAAGGCCCTCAGA-3′, reverse 5′-AGCTTTCCCTCCGCATTGA-3′ (67); *Tnf*, forward 5′-CATCTTCTCAAAATTCGAGTGACAA-3′, reverse 5′-TGGGAGTAGACAAGGTACAACCC-3′ (68); *Ifnb*, forward 5′-AACCTCACCTACAGGGC-3′, reverse 5′-CATTCTGGAGCATCTCTTGG-3′ (42); *Actb*, forward 5′-CCCTGAAGTACCCCATTGAAC-3′, reverse 5′-CTTTTCACGGTTGGCCTTAG-3′. All RT-qPCR assays were performed in duplicate, and the relative expression (rel. expr.) of each gene was determined after normalization to *Actb* transcript levels.

### Data availability.

Both sequencing reads for *M. sympodialis* virus-infected strain KS012 and *M. sympodialis* virus-cured strain SEC494 are available in the NCBI SRA database (BioProject PRJNA593722). The large and small dsRNA fragments of the mycovirus genome have been deposited in GenBank under accession numbers MN812428 and MN831678, respectively.
