# Supplementary figures and images for: A Novel Mycovirus Evokes Transcriptional Rewiring in the Fungus Malassezia and Stimulates Beta Interferon Production in Macrophages
Source: mBio. 2020 Sep 1;11(5):e01534-20. doi: 10.1128/mBio.01534-20 (PMC7468202; doi:10.1128/mBio.01534-20)

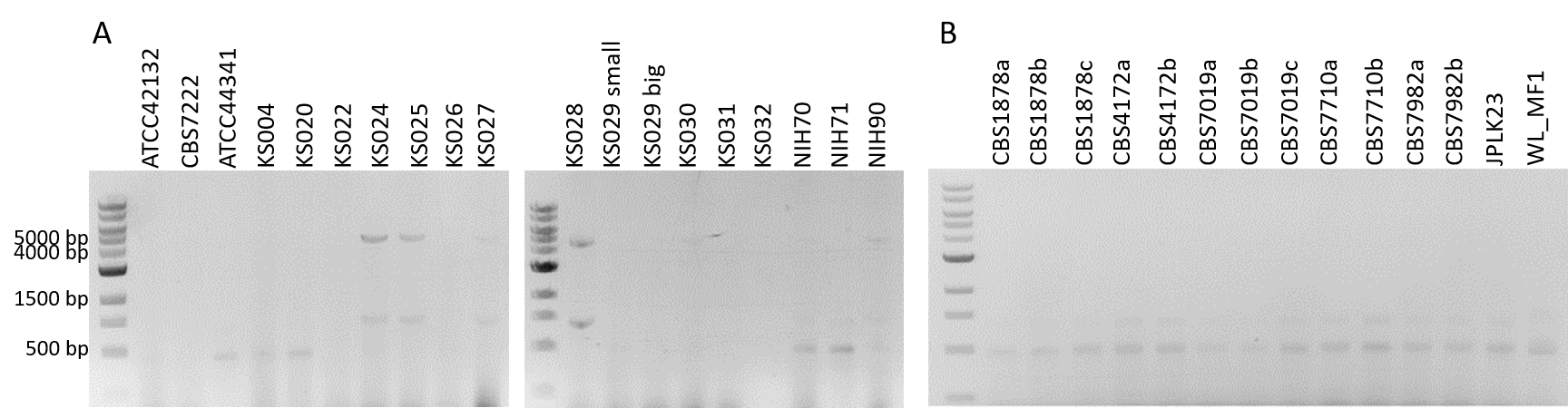

Supplement: FIG S1 [file mBio.01534-20-sf001.tif]

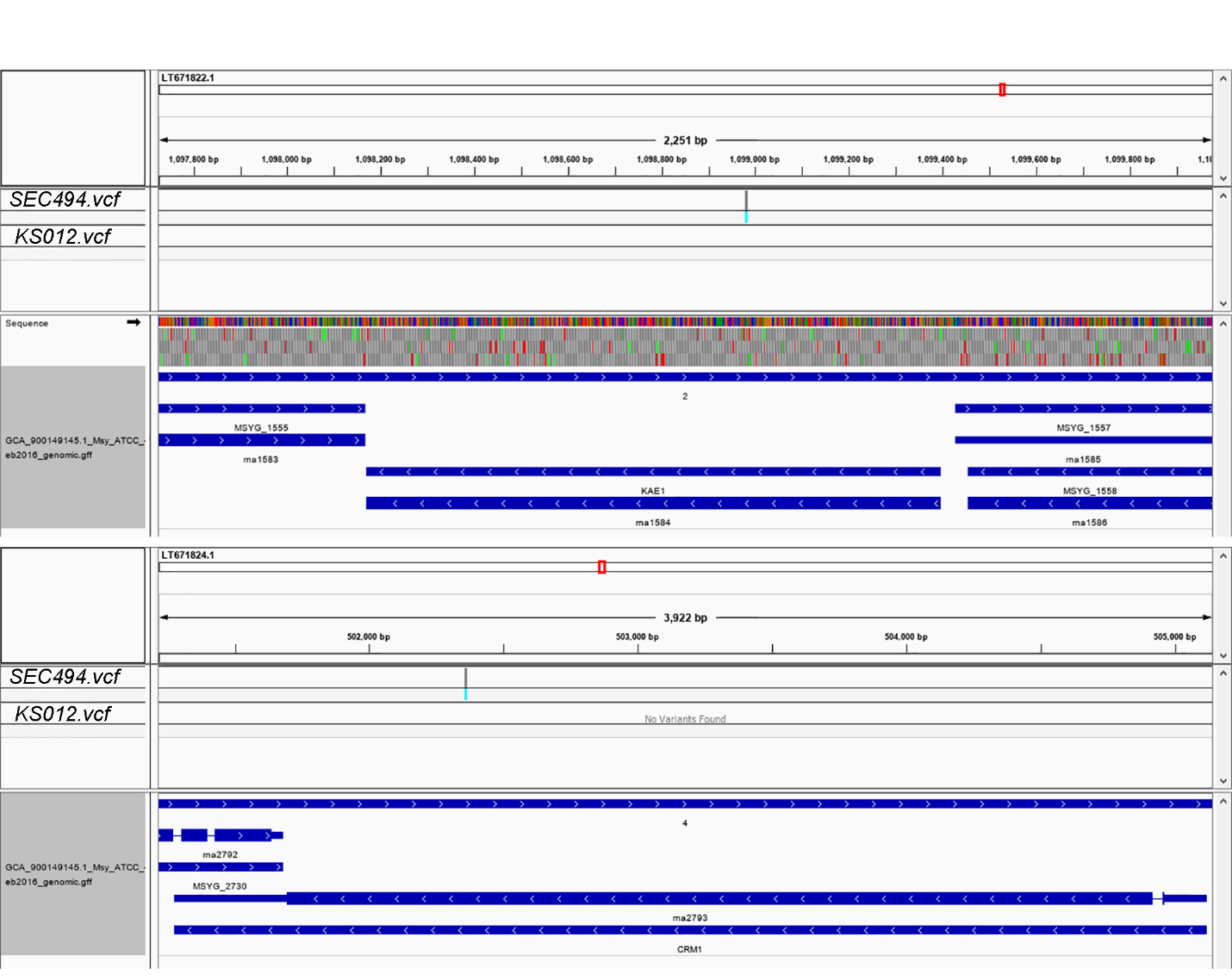

Supplement: TEXT S3 [file mBio.01534-20-s0003.docx]

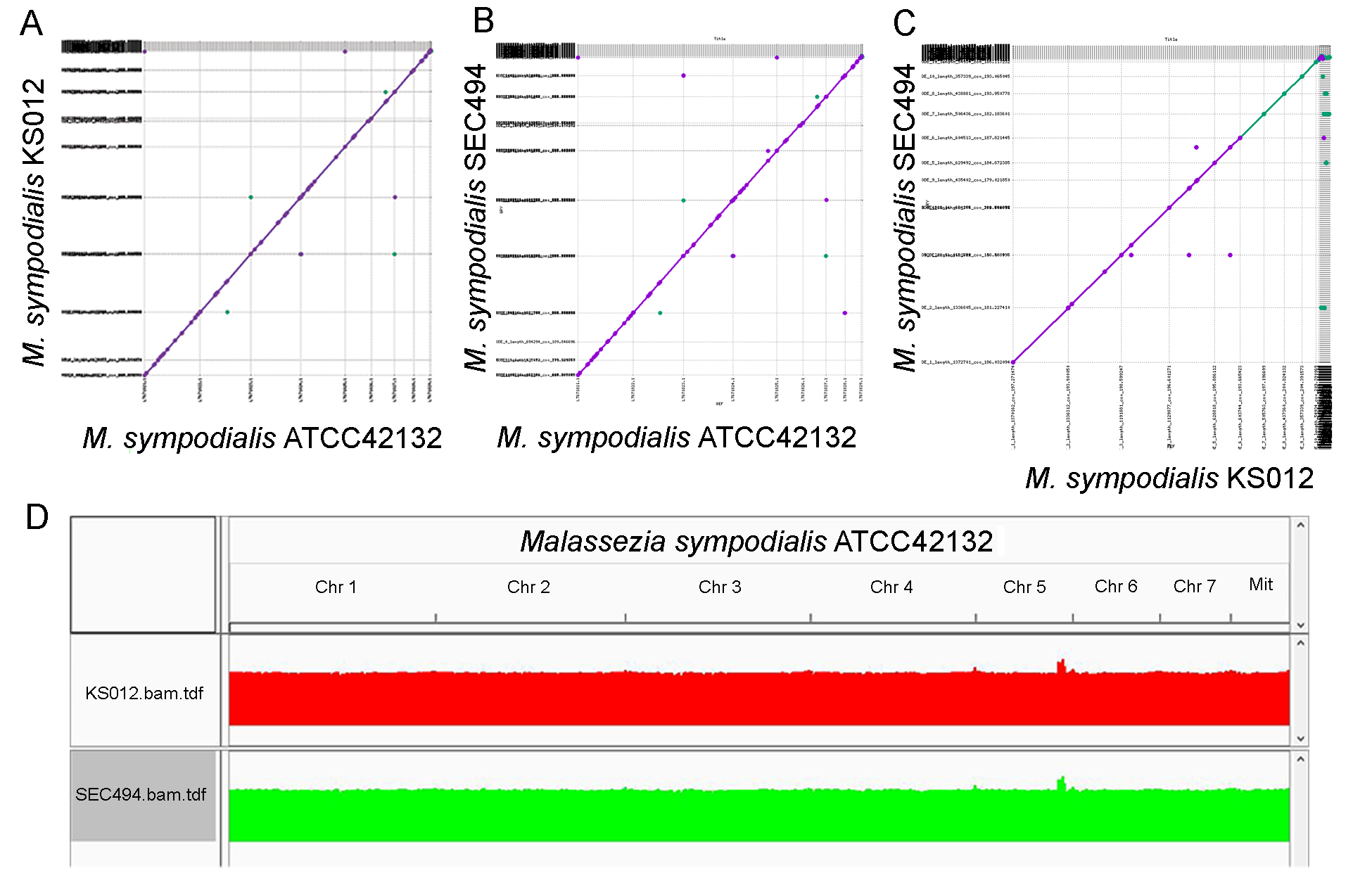

Supplement: FIG S2 [file mBio.01534-20-sf002.tif]

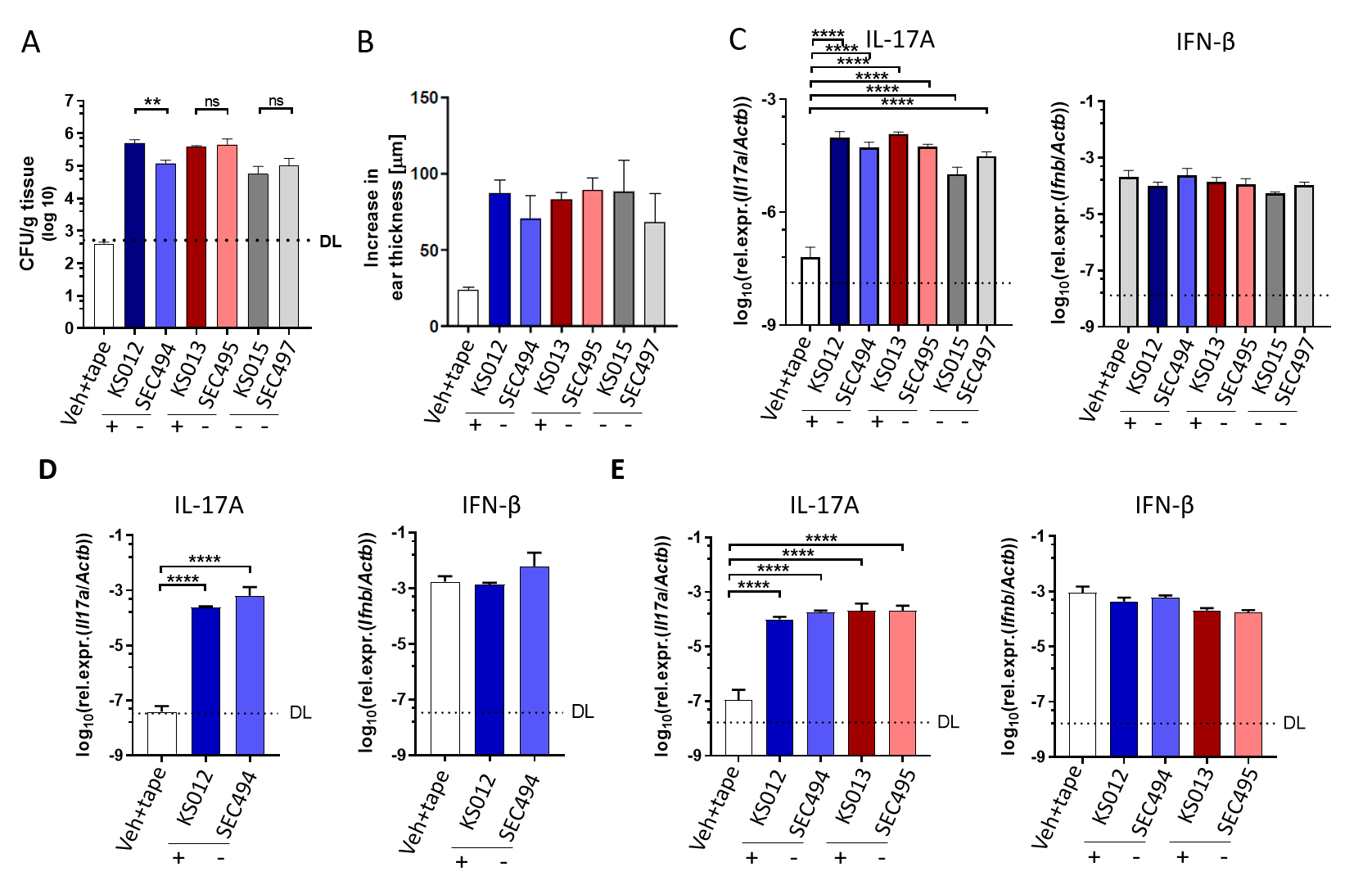

Supplement: FIG S3 [file mBio.01534-20-sf003.tif]

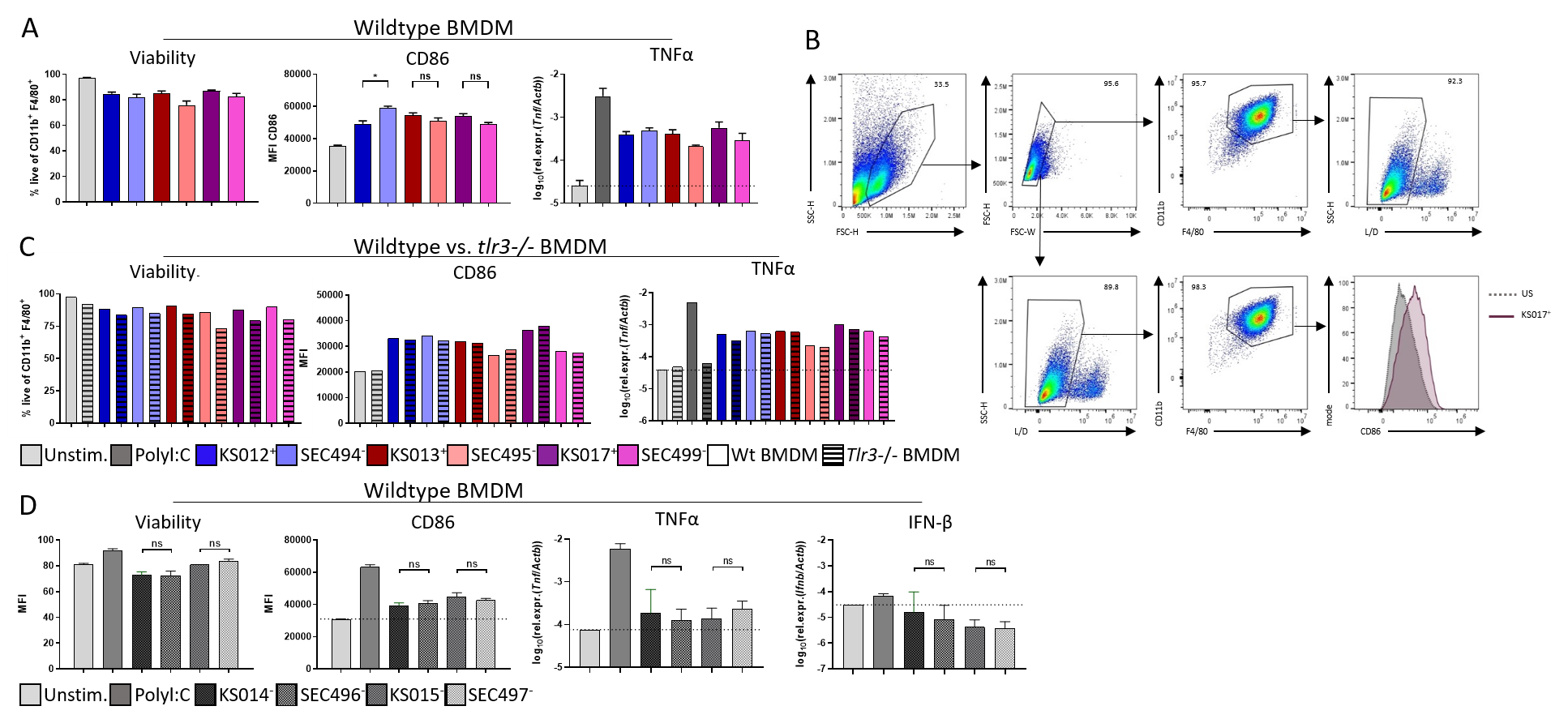

Supplement: FIG S4 [file mBio.01534-20-sf004.tif]
