# Supplementary material for: A Novel Mycovirus Evokes Transcriptional Rewiring in the Fungus Malassezia and Stimulates Beta Interferon Production in Macrophages
Source: mBio. 2020 Sep 1;11(5):e01534-20. doi: 10.1128/mBio.01534-20 (PMC7468202; doi:10.1128/mBio.01534-20)
Supplement: TEXT S1 [file mBio.01534-20-s0001.docx]

>TA-cloning Large fragment (used as query for blastn)

CGATATTTTGATACGCTGGATCAATCCCAAAGATGAGTTTTAATCTTCTGGACCAACTTTGTGGCCCTATGGCTAAAGTGGTTGCTGATTATGCTACTTTAACTAGAAATGTTAAATATGGGATTAAAGCAGATGTCGGATTGAACGTTACTGGTGAGCATACTTACCAGTCAGTGCGTCGTGGCGCCTGTTTTACAAACATGGTCAGCCCGTTTGGTTTTCTCAAGGCCATTATGATTGATGAAACACCAAACTTTTCTGGCACTAATGTGAGGTTCATTAATGACAGTGGTCGTATCGATGAGCGCTGTGTCTATGACACTCTTAAGCTGCAAAGTTACTTCCGCACTTCTGTCATTAACAGTATTGCACCTCTCATTGCTACCATCGGTGGCGAAAACCACGTTTCGGTCCTCGTCAACATGCTCCGCGTATCACGTATTCTGAAATCTAACCCAAAGGAATTCGAAGTTAAGCCTGACGACCTGTTTTACGACGATGGTCATCTAAACATCTCGTACCGCAATTTCCTCAAGCGCGACTTTGAAAGTTTCACCGGATTACGTTTTCAGCCTACTCTCCTCGTTAACCGTTTTGATGACAATGTTGGTTATCCATCTAACATATACTGTCCTAACCTAGAAAACCTGACTTCACGTGAATTTAACATCTTCTGTACTCTGATCTGTGAAATCAACTGTAACTACCCACTTCGTATTGCTTTTAGCTCTCCTTCTCTTGTGGATACATTGTACTTGCCCGGCCATTCGAAGCATTCCGTTAACTATGATCTTGTCAACAACATGACAGCGACTGAAGTTGACATTGTGCTGCGTAAGTATGCGTTTGCGAACAGGGTGGCTAGTGATTTCGATTTGGCTTACTTGATTGTTGTTAACGCGATGTATGCTCCACTGCCACGTGCTGCTGAAGCGCATGGATGGCTGTCACCGGTTAATAACATCTACCTACCAAAGGTTGAAAGTGTTCGTGGTATCATTCCGCAGCTGACTGAAGGTTCACCTTATGAGCCACATCCAGACAGGTTGCTTACTTGGGCTGGTTATTCCAACAATCCTGGTAGGATGATGATTCATGCTCTGGCAACAATCGAGGCCTTCTACACTGGATTATTCGAAATTCTGACTGCGAATCCTCATGGTGTAGAACAATCTCTGAATGCTCTGGGTATGACAAGCTACACATCAGCGAAACCTTACAGGATGTTTTGTGAGGCAGTATCATATCGCTTTGGCAAAGAATTTGACCTACTGTGGAATACGAATGCTGGTGTCGACTGTTATTCTCATTTA

Blastn against the transcriptome of the virus-infected *M. sympodialis* KS012 strain finds two hits:

1) TRINITY_DN8321_c1_g1_i1 len=4613 path=[4591:0-4612] [-1, 4591, -2] Score 2414 **E-value 0.0**

2) TRINITY_DN4986_c1_g1_i1 len=4601 path=[4579:0-4600] [-1, 4579, -2] Score 2412 **E- value 0.0**

**1) HIT 1**

**>TRINITY_DN8321_c1_g1_i1 len=4613 path=[4591:0-4612] [-1, 4591, -2]**

In red is shown the matching sequence following blastn

TGATTGATGAACGATATTTTGATACGCTGGATCAATCCCAAAGATGAGTTTTAATCTTCTGGACCAACTTTGTGGCCCTATGGCTAAAGTGGTTGCTGATTATGCTACTTTAACTAGAAATGTTAAATATGGGATTAAAGCAGATGTCGGATTGAACGTTACTGGTGAGCATACTTACCAGTCAGTGCGTCGTGGCGCCTGTTTTACAAACATGGTCAGCCCGTTTGGTTTTCTCAAGGCCATTATGATTGATGAAACACCAAACTTTTCTGGCACTAATGTGAGGTTCATTAATGACAGTGGTCGTATCGATGAGCGCTGTGTCTATGACACTCTTAAGCTGCAAAGTTACTTCCGCACTTCTGTCATTAACAGTATTGCACCTCTCATTGCTACCATCGGTGGCGAAAACCACGTTTCGGTCCTCGTCAACATGCTCCGCGTATCACGTATTCTGAAATCTAACCCAAAGGAATTCGAAGTTAAGCCTGACGACCTGTTTTACGACGATGGTCATCTAAACATCTCGTACCGCAATTTCCTCAAGCGCGACTTTGAAAGTTTCACCGGATTACGTTTTCAGCCTACTCTCCTCGTTAACCGTTTTGATGACAATGTTGGTTATCCATCTAACATATACTGTCCTAACCTAGAAAACCTGACTTCACGTGAATTTAACATCTTCTGTACTCTGATCTGTGAAATCAACTGTAACTACCCACTTCGTATTGCTTTTAGCTCTCCTTCTCTTGTGGATACATTGTACTTGCCCGGCCATTCGAAGCATTCCGTTAACTATGATCTTGTCAACAACATGACAGCGACTGAAGTTGACATTGTGCTGCGTAAGTATGTGTTTGCGAACAGGGTGGCTAGTGATTTCGATTTGGCTTACTTGATTGTTGTTAACGCGATGTATGCTCCACTGCCACGTGCTGCTGAAGCGCATGGATGGCTGTCACCGGTTAATAACATCTACCTACCAAAGGTTGAAAGTGTTCGTGGTATCATTCCGCAGCTGACTGAAGGTTCGCCTTATGAGCCACATCCAGACAGGTTGCTTACTTGGGCTGGTTATTCCAACAATCCTGGTAGGATGATGATTCATGCTCTGGCAACAATCGAGGCCTTCTACACTGGATTATTCGAAATTCTGACTGCGAATCCTCATGGTGTAGAACAATCTCTGAATGCTCTGGGTATGACAAGCTACACATCAGCGAAACCTTACAGGATGTTTTGTGAGGCAGTATCATATCGCTTTGGCAAAGAATTTGACCTACTGTGGAATACGAATGCTGGTGTCGACTGTTATTCTCATTTACTTGCTACAGAACCAGTGCAACTTGAAGTTACTGCATCATTAGCAGATAACAAGATAGATAACTATGAAGTTTATGAGACTAGCGCACATGGCGAACGTACTGTTCACCTTGTATGTCGTGAGTTAAAACCAGCTCTCTTCCCTATCCTATCAATGGGCATTAACTCAGACCGCTACTTTAACAACTCATTGGAATATGAGACAACGCTTCATTATAATGGTGCTCTTAATGAACTATCTACAACTAGTTCTGATGATGCAAACAAAGCAATGTCTATTTTCCGAGTGGGTGGTTGGAATGCAACACTGGTTGATGCTTCAACGAATAGGGCGATGCGTAACTGGGCTGCTAATTCAAACGGCCAAGTTGTACCTGTTTTACCGCCTGGTATAAATGGGGCCACAACATACAAATTCCCTCTGCGCCTTCTCAACAAAAGGACCCACAGCTGGATGGATATTCCAAATGTAATAAATAAGTTACATATGACAGCAAAGATTGATATTAAAGGCTGGGTCATCATGCTAAACGGTAAGCATGTAGGAGGCTTCATGCCACAGTATAGACCCGTCACGGTCTATCCTAAAGCATTGAATGCTGAGCAGATGACTGCTATACCAATGAAGACATCACCGTCTGCATTGAAGTATAGATTCCAGGGTTTTACACTGGCTGCGAGTCAAGAAAATGCACCCGTTGTCCATTTACAATCATCGCATATGGACACGGCCGACTTGCAGCCAGTCGAGGACTTTCAAGATCCGGCTGGTGCTCCGGAGGAAGCCTGAACTCTAACTACCATGATTTGAGTAGTTATCATAACCCGTTGCCCTTGTACCTTGACGAAAAGTATGATAACTCGTTGCCTGAGAAGTGTTCGTATATTCTTATGTATGTATCCAACGATGATGTGCCTTCACATGTTGTTGATTACCCATTCGAACTCCCTATCACTAAAGAAAGAGTGTGGGGTTTGCGGATTCGTCGTCGCAATCTGACGTGTTGGTACGTTGCTACCATGTCAATTTCGCACTACACACCGGCTACTATGTCGATTGCAGGCGGAATGATTTCTGGTAACCTTCGTGCGGGTCTTACTTTCGAGAATCATGTCGACTACTACTCACACATCCCTGAGATTGATGAACACCTTAAGGTCATATCACCTCAAGAGTTGTTTGATTTCGTGGCGGCGTTGCCGAAGACTAAAATCACGCAGCAGCATCATCTTTACGTCAACAAGCAAGAGTTAATAGTGTGTTATGAAAAGTTCCTGGCTGAATCCAGTGCAGAGAAAATCACAGCTGTAAACGAAACAATAAACCTTATGATTAAGAAATGCGAAAAACTTGGAATTGACAATAGAGCTTCATTTAATACTCATGTTATGTATGTATTACTAGCCCCTATATTTGCAGTCTATCATCTGCTTGAAATTATGAGAAATACAAACAACACAAATGAATACTTCAATTTACTTAAACTCGAAAGTGGAGCAGCCAAACAGTCACAGACTGTGTTCAGAAATGACCTTGCAGTCATTTACGAGATGCAAGTACTCTTCAACAGAGTACACGCCGATGTGGATTGGAAAACTGAGAAACAGCATCGTACTGAATCGAAGACAGTACCTATCCCATGCGAAGTAGTTTATTCACTAGCGACCTCGATTTTCAAATCTGGTATCGCAGAAGGCCGTGCACCTTTGCGTTTGGATTGGAACGGTTACTGGGCTGGAAGGTGGTCATCAATGCCTAATGGCTCAATTGTAAGCCAGTACGAAACTGACTTAGAGTTAAAGCGCTCGCTACCAAAAGAAGCGAAGTACAAGTCGTGCTGGTTTGCAGTGAATGGTCACAGTGATCACAGCTTCTGGGCGAAGCGCAACCCCGAAATTTATGCTACTACAAGCACTAAGTATGAATGGGGCAAAGTGCGCGCTCTGTACGGCTGTGATGTTACTTCATTTCTTCATGCAGACTTCGCGATGAGCAACTGTGAAGATACCCTGCCTTCCTGTTTCCCTGTAGGCAAGTTCGCAACAAAGAGCTTTGTCGAAGGCAGTGTGCACAAGTTTAAAGACACTGTGCCGGTATGCTTCGATTATGATGACTTCAACAGTCAGCACTCAATTGCTAGTATGCAAGCAGTCATGCGTGCTTGGATTGATGTCTATAGTACCTTTCTAACTGAGGAACAGAAGATATCAGCTGAGTGGACGCATGACTCGCTTGCGAATATGAGTGTATGCTTCAACGCGTTGGGTGAGACTGTCACTATTGATGGTACTCTCATGAGCGGCTGGCGTCTTACATCTTACATGAACACAGTGCTGAATCGTGTCTATCTACTACATGCAGGTTTAGACAAGCTACTTGTATATTCTTTGCATAATGGTGATGACATGTTCGGCGGTGCTCCTAATCTGCACAATGCGCTGCAGCTAATCAAGAATGCTAAAGAACGTGGCATTCGCGCCCAGGTCAGTAAGACTAACCTGGGCACTATTGGCGAATTCCTTCGCGTCGACACGCGCGCAAAGGATTCACAGATGACGCAGTATCTGGCCCGCGCCGTCTCAACGCTGGTTCACGGCCGTGTTGAGGCTGATTCACCCACTGATCTAGTGGCCTTCATCACCGCTACGATCACACGTGTTGAAGAGGTGAAGTCCCGCGGCGGTAGTGCCACTGTGCTAGACGCATTGTTCAACAAGATTATGGAGTTTGCTTCTAAGTTATTCAGTACTGATCCAGAAGTAATTGAAGCTATTCTCACAACGCATCCCACTCAAGGTGGTGTTAATAAAGACGCTCCTGTACGAGCAAAGTACCTGAAGCGGAAGGCACACTCCGGCAAGGATGATATCTACTACCATCAGAAGTACTCTATTCTCGGGGACGGAATTAATGATTACATTAATCATGTAAAGCAGAGATTCCATCTCCGAGAAAGTGAGCTAGATCGCAGTAGACTCCAGCTGAAGGCCTACCAGTCTTTGGAACGCGATCTTGTTAGTTACGAGACCGGAATCGAAACTAATGAGAGAATCGCAATCTACAGAGGTCTAAGTGGTGCATGGAAGGGCACAGGTTTTGAAGCACCTATTGCTAAAGTCAGATCGATGGGTCTCATTGCAGCCAAGCAACTGAGAGTGCTGACATCTACTTTAGCTAGGATGATTCAGAATGCTGATGACCCTATTACTTTCATGCGAGTAGTCACTTAGACCTCTGTATAAATCGTTCTGCAGCTTTAGGCTGGCAGAACGCCGTGCGTTATTATATA

**2) HIT 2**

**>TRINITY_DN4986_c1_g1_i1 len=4601 path=[4579:0-4600] [-1, 4579, -2]** (displayed in reverse complement orientation)

In red is shown the matching sequence following blastn

CCCCGGGATATTTTGATACGCTGGATCAATCCCAAAGATGAGTTTTAATCTTCTGGACCAACTTTGTGGCCCTATGGCTAAAGTGGTTGCTGATTATGCTACTTTAACTAGAAATGTTAAATATGGGATTAAAGCAGATGTCGGATTGAACGTTACTGGTGAGCATACTTACCAGTCAGTGCGTCGTGGCGCCTGTTTTACAAACATGGTCAGCCCGTTTGGTTTTCTCAAGGCCATTATGATTGATGAAACACCAAACTTTTCTGGCACTAATGTGAGGTTCATTAATGACAGTGGTCGTATCGATGAGCGCTGTGTCTATGACACTCTTAAGCTGCAAAGTTACTTCCGCACTTCTGTCATTAACAGTATTGCACCTCTCATTGCTACCATCGGTGGCGAAAACCACGTTTCGGTCCTCGTCAACATGCTCCGCGTATCACGTATTCTGAAATCTAACCCAAAGGAATTCGAAGTTAAGCCTGACGACCTGTTTTACGACGATGGTCATCTAAACATCTCGTACCGCAATTTCCTCAAGCGCGACTTTGAAAGTTTCACCGGATTACGTTTTCAGCCTACTCTCCTCGTTAACCGTTTTGATGACAATGTTGGTTATCCATCTAACATATACTGTCCTAACCTAGAAAACCTGACTTCACGTGAATTTAACATCTTCTGTACTCTGATCTGTGAAATCAACTGTAACTACCCACTTCGTATTGCTTTTAGCTCTCCTTCTCTTGTGGATACATTGTACTTGCCCGGCCATTCGAAGCATTCCGTTAACTATGATCTTGTCAACAACATGACAGCGACTGAAGTTGACATTGTGCTGCGTAAGTATGTGTTTGCGAACAGGGTGGCTAGTGATTTCGATTTGGCTTACTTGATTGTTGTTAACGCGATGTATGCTCCACTGCCACGTGCTGCTGAAGCGCATGGATGGCTGTCACCGGTTAATAACATCTACCTACCAAAGGTTGAAAGTGTTCGTGGTATCATTCCGCAGCTGACTGAAGGTTCGCCTTATGAGCCACATCCAGACAGGTTGCTTACTTGGGCTGGTTATTCCAACAATCCTGGTAGGATGATGATTCATGCTCTGGCAACAATCGAGGCCTTCTACACTGGATTATTCGAAATTCTGACTGCGAATCCTCATGGTGTAGAACAATCTCTGAATGCTCTGGGTATGACAAGCTACACATCAGCGAAACCTTACAGGATGTTTTGTGAGGCAGTATCATATCGCTTTGGCAAAGAATTTGACCTACTGTGGAATACGAATGCTGGTGTCGACTGTTATTCTCATTTACTTGCTACAGAACCAGTGCAACTTGAAGTTACTGCATCATTAGCAGATAACAAGATAGATAACTATGAAGTTTATGAGACTAGCGCACATGGCGAACGTACTGTTCACCTTGTATGTCGTGAGTTAAAACCAGCTCTCTTCCCTATCCTATCAATGGGCATTAACTCAGACCGCTACTTTAACAACTCATTGGAATATGAGACAACGCTTCATTATAATGGTGCTCTTAATGAACTATCTACAACTAGTTCTGATGATGCAAACAAAGCAATGTCTATTTTCCGAGTGGGTGGTTGGAATGCAACACTGGTTGATGCTTCAACGAATAGGGCGATGCGTAACTGGGCTGCTAATTCAAACGGCCAAGTTGTACCTGTTTTACCGCCTGGTATAAATGGGGCCACAACATACAAATTCCCTCTGCGCCTTCTCAACAAAAGGACCCACAGCTGGATGGATATTCCAAATGTAATAAATAAGTTACATATGACAGCAAAGATTGATATTAAAGGCTGGGTCATCATGCTAAACGGTAAGCATGTAGGAGGCTTCATGCCACAGTATAGACCCGTCACGGTCTATCCTAAAGCATTGAATGCTGAGCAGATGACTGCTATACCAATGAAGACATCACCGTCTGCATTGAAGTATAGATTCCAGGGTTTTACACTGGCTGCGAGTCAAGAAAATGCACCCGTTGTCCATTTACAATCATCGCATATGGACACGGCCGACTTGCAGCCAGTCGAGGACTTTCAAGATCCGGCTGGTGCTCCGGAGGAAGCCTGAACTCTAACTACCATGATTTGAGTAGTTATCATAACCCGTTGCCCTTGTACCTTGACGAAAAGTATGATAACTCGTTGCCTGAGAAGTGTTCGTATATTCTTATGTATGTATCCAACGATGATGTGCCTTCACATGTTGTTGATTACCCATTCGAACTCCCTATCACTAAAGAAAGAGTGTGGGGTTTGCGGATTCGTCGTCGCAATCTGACGTGTTGGTACGTTGCTACCATGTCAATTTCGCACTACACACCGGCTACTATGTCGATTGCAGGCGGAATGATTTCTGGTAACCTTCGTGCGGGTCTTACTTTCGAGAATCATGTCGACTACTACTCACACATCCCTGAGATTGATGAACACCTTAAGGTCATATCACCTCAAGAGTTGTTTGATTTCGTGGCGGCGTTGCCGAAGACTAAAATCACGCAGCAGCATCATCTTTACGTCAACAAGCAAGAGTTAATAGTGTGTTATGAAAAGTTCCTGGCTGAATCCAGTGCAGAGAAAATCACAGCTGTAAACGAAACAATAAACCTTATGATTAAGAAATGCGAAAAACTTGGAATTGACAATAGAGCTTCATTTAATACTCATGTTATGTATGTATTACTAGCCCCTATATTTGCAGTCTATCATCTGCTTGAAATTATGAGAAATACAAACAACACAAATGAATACTTCAATTTACTTAAACTCGAAAGTGGAGCAGCCAAACAGTCACAGACTGTGTTCAGAAATGACCTTGCAGTCATTTACGAGATGCAAGTACTCTTCAACAGAGTACACGCCGATGTGGATTGGAAAACTGAGAAACAGCATCGTACTGAATCGAAGACAGTACCTATCCCATGCGAAGTAGTTTATTCACTAGCGACCTCGATTTTCAAATCTGGTATCGCAGAAGGCCGTGCACCTTTGCGTTTGGATTGGAACGGTTACTGGGCTGGAAGGTGGTCATCAATGCCTAATGGCTCAATTGTAAGCCAGTACGAAACTGACTTAGAGTTAAAGCGCTCGCTACCAAAAGAAGCGAAGTACAAGTCGTGCTGGTTTGCAGTGAATGGTCACAGTGATCACAGCTTCTGGGCGAAGCGCAACCCCGAAATTTATGCTACTACAAGCACTAAGTATGAATGGGGCAAAGTGCGCGCTCTGTACGGCTGTGATGTTACTTCATTTCTTCATGCAGACTTCGCGATGAGCAACTGTGAAGATACCCTGCCTTCCTGTTTCCCTGTAGGCAAGTTCGCAACAAAGAGCTTTGTCGAAGGCAGTGTGCACAAGTTTAAAGACACTGTGCCGGTATGCTTCGATTATGATGACTTCAACAGTCAGCACTCAATTGCTAGTATGCAAGCAGTCATGCGTGCTTGGATTGATGTCTATAGTACCTTTCTAACTGAGGAACAGAAGATATCAGCTGAGTGGACGCATGACTCGCTTGCGAATATGAGTGTATGCTTCAACGCGTTGGGTGAGACTGTCACTATTGATGGTACTCTCATGAGCGGCTGGCGTCTTACATCTTACATGAACACAGTGCTGAATCGTGTCTATCTACTACATGCAGGTTTAGACAAGCTACTTGTATATTCTTTGCATAATGGTGATGACATGTTCGGCGGTGCTCCTAATCTGCACAATGCGCTGCAGCTAATCAAGAATGCTAAAGAACGTGGCATTCGCGCCCAGGTCAGTAAGACTAACCTGGGCACTATTGGCGAATTCCTTCGCGTCGACACGCGCGCAAAGGATTCACAGATGACGCAGTATCTGGCCCGCGCCGTCTCAACGCTGGTTCACGGCCGTGTTGAGGCTGATTCACCCACTGATCTAGTGGCCTTCATCACCGCTACGATCACACGTGTTGAAGAGGTGAAGTCCCGCGGCGGTAGTGCCACTGTGCTAGACGCATTGTTCAACAAGATTATGGAGTTTGCTTCTAAGTTATTCAGTACTGATCCAGAAGTAATTGAAGCTATTCTCACAACGCATCCCACTCAAGGTGGTGTTAATAAAGACGCTCCTGTACGAGCAAAGTACCTGAAGCGGAAGGCACACTCCGGCAAGGATGATATCTACTACCATCAGAAGTACTCTATTCTCGGGGACGGAATTAATGATTACATTAATCATGTAAAGCAGAGATTCCATCTCCGAGAAAGTGAGCTAGATCGCAGTAGACTCCAGCTGAAGGCCTACCAGTCTTTGGAACGCGATCTTGTTAGTTACGAGACCGGAATCGAAACTAATGAGAGAATCGCAATCTACAGAGGTCTAAGTGGTGCATGGAAGGGCACAGGTTTTGAAGCACCTATTGCTAAAGTCAGATCGATGGGTCTCATTGCAGCCAAGCAACTGAGAGTGCTGACATCTACTTTAGCTAGGATGATTCAGAATGCTGATGACCCTATTACTTTCATGCGAGTAGTCACTTAGACCTCTGTATAAATCGTTCTGCAGCTTTAGGCTGGCAGAACGCCGTGCGTTAT

CLUSTAL O(1.2.4) multiple sequence alignment

TRINITY_DN8321 TGATTGATGAACGATATTTTGATACGCTGGATCAATCCCAAAGATGAGTTTTAATCTTCT 60

TRINITY_DN4986 ------CCCCGGGATATTTTGATACGCTGGATCAATCCCAAAGATGAGTTTTAATCTTCT 54

************************************************

TRINITY_DN8321 GGACCAACTTTGTGGCCCTATGGCTAAAGTGGTTGCTGATTATGCTACTTTAACTAGAAA 120

TRINITY_DN4986 GGACCAACTTTGTGGCCCTATGGCTAAAGTGGTTGCTGATTATGCTACTTTAACTAGAAA 114

************************************************************

TRINITY_DN8321 TGTTAAATATGGGATTAAAGCAGATGTCGGATTGAACGTTACTGGTGAGCATACTTACCA 180

TRINITY_DN4986 TGTTAAATATGGGATTAAAGCAGATGTCGGATTGAACGTTACTGGTGAGCATACTTACCA 174

************************************************************

TRINITY_DN8321 GTCAGTGCGTCGTGGCGCCTGTTTTACAAACATGGTCAGCCCGTTTGGTTTTCTCAAGGC 240

TRINITY_DN4986 GTCAGTGCGTCGTGGCGCCTGTTTTACAAACATGGTCAGCCCGTTTGGTTTTCTCAAGGC 234

************************************************************

TRINITY_DN8321 CATTATGATTGATGAAACACCAAACTTTTCTGGCACTAATGTGAGGTTCATTAATGACAG 300

TRINITY_DN4986 CATTATGATTGATGAAACACCAAACTTTTCTGGCACTAATGTGAGGTTCATTAATGACAG 294

************************************************************

TRINITY_DN8321 TGGTCGTATCGATGAGCGCTGTGTCTATGACACTCTTAAGCTGCAAAGTTACTTCCGCAC 360

TRINITY_DN4986 TGGTCGTATCGATGAGCGCTGTGTCTATGACACTCTTAAGCTGCAAAGTTACTTCCGCAC 354

************************************************************

TRINITY_DN8321 TTCTGTCATTAACAGTATTGCACCTCTCATTGCTACCATCGGTGGCGAAAACCACGTTTC 420

TRINITY_DN4986 TTCTGTCATTAACAGTATTGCACCTCTCATTGCTACCATCGGTGGCGAAAACCACGTTTC 414

************************************************************

TRINITY_DN8321 GGTCCTCGTCAACATGCTCCGCGTATCACGTATTCTGAAATCTAACCCAAAGGAATTCGA 480

TRINITY_DN4986 GGTCCTCGTCAACATGCTCCGCGTATCACGTATTCTGAAATCTAACCCAAAGGAATTCGA 474

************************************************************

TRINITY_DN8321 AGTTAAGCCTGACGACCTGTTTTACGACGATGGTCATCTAAACATCTCGTACCGCAATTT 540

TRINITY_DN4986 AGTTAAGCCTGACGACCTGTTTTACGACGATGGTCATCTAAACATCTCGTACCGCAATTT 534

************************************************************

TRINITY_DN8321 CCTCAAGCGCGACTTTGAAAGTTTCACCGGATTACGTTTTCAGCCTACTCTCCTCGTTAA 600

TRINITY_DN4986 CCTCAAGCGCGACTTTGAAAGTTTCACCGGATTACGTTTTCAGCCTACTCTCCTCGTTAA 594

************************************************************

TRINITY_DN8321 CCGTTTTGATGACAATGTTGGTTATCCATCTAACATATACTGTCCTAACCTAGAAAACCT 660

TRINITY_DN4986 CCGTTTTGATGACAATGTTGGTTATCCATCTAACATATACTGTCCTAACCTAGAAAACCT 654

************************************************************

TRINITY_DN8321 GACTTCACGTGAATTTAACATCTTCTGTACTCTGATCTGTGAAATCAACTGTAACTACCC 720

TRINITY_DN4986 GACTTCACGTGAATTTAACATCTTCTGTACTCTGATCTGTGAAATCAACTGTAACTACCC 714

************************************************************

TRINITY_DN8321 ACTTCGTATTGCTTTTAGCTCTCCTTCTCTTGTGGATACATTGTACTTGCCCGGCCATTC 780

TRINITY_DN4986 ACTTCGTATTGCTTTTAGCTCTCCTTCTCTTGTGGATACATTGTACTTGCCCGGCCATTC 774

************************************************************

TRINITY_DN8321 GAAGCATTCCGTTAACTATGATCTTGTCAACAACATGACAGCGACTGAAGTTGACATTGT 840

TRINITY_DN4986 GAAGCATTCCGTTAACTATGATCTTGTCAACAACATGACAGCGACTGAAGTTGACATTGT 834

************************************************************

TRINITY_DN8321 GCTGCGTAAGTATGTGTTTGCGAACAGGGTGGCTAGTGATTTCGATTTGGCTTACTTGAT 900

TRINITY_DN4986 GCTGCGTAAGTATGTGTTTGCGAACAGGGTGGCTAGTGATTTCGATTTGGCTTACTTGAT 894

************************************************************

TRINITY_DN8321 TGTTGTTAACGCGATGTATGCTCCACTGCCACGTGCTGCTGAAGCGCATGGATGGCTGTC 960

TRINITY_DN4986 TGTTGTTAACGCGATGTATGCTCCACTGCCACGTGCTGCTGAAGCGCATGGATGGCTGTC 954

************************************************************

TRINITY_DN8321 ACCGGTTAATAACATCTACCTACCAAAGGTTGAAAGTGTTCGTGGTATCATTCCGCAGCT 1020

TRINITY_DN4986 ACCGGTTAATAACATCTACCTACCAAAGGTTGAAAGTGTTCGTGGTATCATTCCGCAGCT 1014

************************************************************

TRINITY_DN8321 GACTGAAGGTTCGCCTTATGAGCCACATCCAGACAGGTTGCTTACTTGGGCTGGTTATTC 1080

TRINITY_DN4986 GACTGAAGGTTCGCCTTATGAGCCACATCCAGACAGGTTGCTTACTTGGGCTGGTTATTC 1074

************************************************************

TRINITY_DN8321 CAACAATCCTGGTAGGATGATGATTCATGCTCTGGCAACAATCGAGGCCTTCTACACTGG 1140

TRINITY_DN4986 CAACAATCCTGGTAGGATGATGATTCATGCTCTGGCAACAATCGAGGCCTTCTACACTGG 1134

************************************************************

TRINITY_DN8321 ATTATTCGAAATTCTGACTGCGAATCCTCATGGTGTAGAACAATCTCTGAATGCTCTGGG 1200

TRINITY_DN4986 ATTATTCGAAATTCTGACTGCGAATCCTCATGGTGTAGAACAATCTCTGAATGCTCTGGG 1194

************************************************************

TRINITY_DN8321 TATGACAAGCTACACATCAGCGAAACCTTACAGGATGTTTTGTGAGGCAGTATCATATCG 1260

TRINITY_DN4986 TATGACAAGCTACACATCAGCGAAACCTTACAGGATGTTTTGTGAGGCAGTATCATATCG 1254

************************************************************

TRINITY_DN8321 CTTTGGCAAAGAATTTGACCTACTGTGGAATACGAATGCTGGTGTCGACTGTTATTCTCA 1320

TRINITY_DN4986 CTTTGGCAAAGAATTTGACCTACTGTGGAATACGAATGCTGGTGTCGACTGTTATTCTCA 1314

************************************************************

TRINITY_DN8321 TTTACTTGCTACAGAACCAGTGCAACTTGAAGTTACTGCATCATTAGCAGATAACAAGAT 1380

TRINITY_DN4986 TTTACTTGCTACAGAACCAGTGCAACTTGAAGTTACTGCATCATTAGCAGATAACAAGAT 1374

************************************************************

TRINITY_DN8321 AGATAACTATGAAGTTTATGAGACTAGCGCACATGGCGAACGTACTGTTCACCTTGTATG 1440

TRINITY_DN4986 AGATAACTATGAAGTTTATGAGACTAGCGCACATGGCGAACGTACTGTTCACCTTGTATG 1434

************************************************************

TRINITY_DN8321 TCGTGAGTTAAAACCAGCTCTCTTCCCTATCCTATCAATGGGCATTAACTCAGACCGCTA 1500

TRINITY_DN4986 TCGTGAGTTAAAACCAGCTCTCTTCCCTATCCTATCAATGGGCATTAACTCAGACCGCTA 1494

************************************************************

TRINITY_DN8321 CTTTAACAACTCATTGGAATATGAGACAACGCTTCATTATAATGGTGCTCTTAATGAACT 1560

TRINITY_DN4986 CTTTAACAACTCATTGGAATATGAGACAACGCTTCATTATAATGGTGCTCTTAATGAACT 1554

************************************************************

TRINITY_DN8321 ATCTACAACTAGTTCTGATGATGCAAACAAAGCAATGTCTATTTTCCGAGTGGGTGGTTG 1620

TRINITY_DN4986 ATCTACAACTAGTTCTGATGATGCAAACAAAGCAATGTCTATTTTCCGAGTGGGTGGTTG 1614

************************************************************

TRINITY_DN8321 GAATGCAACACTGGTTGATGCTTCAACGAATAGGGCGATGCGTAACTGGGCTGCTAATTC 1680

TRINITY_DN4986 GAATGCAACACTGGTTGATGCTTCAACGAATAGGGCGATGCGTAACTGGGCTGCTAATTC 1674

************************************************************

TRINITY_DN8321 AAACGGCCAAGTTGTACCTGTTTTACCGCCTGGTATAAATGGGGCCACAACATACAAATT 1740

TRINITY_DN4986 AAACGGCCAAGTTGTACCTGTTTTACCGCCTGGTATAAATGGGGCCACAACATACAAATT 1734

************************************************************

TRINITY_DN8321 CCCTCTGCGCCTTCTCAACAAAAGGACCCACAGCTGGATGGATATTCCAAATGTAATAAA 1800

TRINITY_DN4986 CCCTCTGCGCCTTCTCAACAAAAGGACCCACAGCTGGATGGATATTCCAAATGTAATAAA 1794

************************************************************

TRINITY_DN8321 TAAGTTACATATGACAGCAAAGATTGATATTAAAGGCTGGGTCATCATGCTAAACGGTAA 1860

TRINITY_DN4986 TAAGTTACATATGACAGCAAAGATTGATATTAAAGGCTGGGTCATCATGCTAAACGGTAA 1854

************************************************************

TRINITY_DN8321 GCATGTAGGAGGCTTCATGCCACAGTATAGACCCGTCACGGTCTATCCTAAAGCATTGAA 1920

TRINITY_DN4986 GCATGTAGGAGGCTTCATGCCACAGTATAGACCCGTCACGGTCTATCCTAAAGCATTGAA 1914

************************************************************

TRINITY_DN8321 TGCTGAGCAGATGACTGCTATACCAATGAAGACATCACCGTCTGCATTGAAGTATAGATT 1980

TRINITY_DN4986 TGCTGAGCAGATGACTGCTATACCAATGAAGACATCACCGTCTGCATTGAAGTATAGATT 1974

************************************************************

TRINITY_DN8321 CCAGGGTTTTACACTGGCTGCGAGTCAAGAAAATGCACCCGTTGTCCATTTACAATCATC 2040

TRINITY_DN4986 CCAGGGTTTTACACTGGCTGCGAGTCAAGAAAATGCACCCGTTGTCCATTTACAATCATC 2034

************************************************************

TRINITY_DN8321 GCATATGGACACGGCCGACTTGCAGCCAGTCGAGGACTTTCAAGATCCGGCTGGTGCTCC 2100

TRINITY_DN4986 GCATATGGACACGGCCGACTTGCAGCCAGTCGAGGACTTTCAAGATCCGGCTGGTGCTCC 2094

************************************************************

TRINITY_DN8321 GGAGGAAGCCTGAACTCTAACTACCATGATTTGAGTAGTTATCATAACCCGTTGCCCTTG 2160

TRINITY_DN4986 GGAGGAAGCCTGAACTCTAACTACCATGATTTGAGTAGTTATCATAACCCGTTGCCCTTG 2154

************************************************************

TRINITY_DN8321 TACCTTGACGAAAAGTATGATAACTCGTTGCCTGAGAAGTGTTCGTATATTCTTATGTAT 2220

TRINITY_DN4986 TACCTTGACGAAAAGTATGATAACTCGTTGCCTGAGAAGTGTTCGTATATTCTTATGTAT 2214

************************************************************

TRINITY_DN8321 GTATCCAACGATGATGTGCCTTCACATGTTGTTGATTACCCATTCGAACTCCCTATCACT 2280

TRINITY_DN4986 GTATCCAACGATGATGTGCCTTCACATGTTGTTGATTACCCATTCGAACTCCCTATCACT 2274

************************************************************

TRINITY_DN8321 AAAGAAAGAGTGTGGGGTTTGCGGATTCGTCGTCGCAATCTGACGTGTTGGTACGTTGCT 2340

TRINITY_DN4986 AAAGAAAGAGTGTGGGGTTTGCGGATTCGTCGTCGCAATCTGACGTGTTGGTACGTTGCT 2334

************************************************************

TRINITY_DN8321 ACCATGTCAATTTCGCACTACACACCGGCTACTATGTCGATTGCAGGCGGAATGATTTCT 2400

TRINITY_DN4986 ACCATGTCAATTTCGCACTACACACCGGCTACTATGTCGATTGCAGGCGGAATGATTTCT 2394

************************************************************

TRINITY_DN8321 GGTAACCTTCGTGCGGGTCTTACTTTCGAGAATCATGTCGACTACTACTCACACATCCCT 2460

TRINITY_DN4986 GGTAACCTTCGTGCGGGTCTTACTTTCGAGAATCATGTCGACTACTACTCACACATCCCT 2454

************************************************************

TRINITY_DN8321 GAGATTGATGAACACCTTAAGGTCATATCACCTCAAGAGTTGTTTGATTTCGTGGCGGCG 2520

TRINITY_DN4986 GAGATTGATGAACACCTTAAGGTCATATCACCTCAAGAGTTGTTTGATTTCGTGGCGGCG 2514

************************************************************

TRINITY_DN8321 TTGCCGAAGACTAAAATCACGCAGCAGCATCATCTTTACGTCAACAAGCAAGAGTTAATA 2580

TRINITY_DN4986 TTGCCGAAGACTAAAATCACGCAGCAGCATCATCTTTACGTCAACAAGCAAGAGTTAATA 2574

************************************************************

TRINITY_DN8321 GTGTGTTATGAAAAGTTCCTGGCTGAATCCAGTGCAGAGAAAATCACAGCTGTAAACGAA 2640

TRINITY_DN4986 GTGTGTTATGAAAAGTTCCTGGCTGAATCCAGTGCAGAGAAAATCACAGCTGTAAACGAA 2634

************************************************************

TRINITY_DN8321 ACAATAAACCTTATGATTAAGAAATGCGAAAAACTTGGAATTGACAATAGAGCTTCATTT 2700

TRINITY_DN4986 ACAATAAACCTTATGATTAAGAAATGCGAAAAACTTGGAATTGACAATAGAGCTTCATTT 2694

************************************************************

TRINITY_DN8321 AATACTCATGTTATGTATGTATTACTAGCCCCTATATTTGCAGTCTATCATCTGCTTGAA 2760

TRINITY_DN4986 AATACTCATGTTATGTATGTATTACTAGCCCCTATATTTGCAGTCTATCATCTGCTTGAA 2754

************************************************************

TRINITY_DN8321 ATTATGAGAAATACAAACAACACAAATGAATACTTCAATTTACTTAAACTCGAAAGTGGA 2820

TRINITY_DN4986 ATTATGAGAAATACAAACAACACAAATGAATACTTCAATTTACTTAAACTCGAAAGTGGA 2814

************************************************************

TRINITY_DN8321 GCAGCCAAACAGTCACAGACTGTGTTCAGAAATGACCTTGCAGTCATTTACGAGATGCAA 2880

TRINITY_DN4986 GCAGCCAAACAGTCACAGACTGTGTTCAGAAATGACCTTGCAGTCATTTACGAGATGCAA 2874

************************************************************

TRINITY_DN8321 GTACTCTTCAACAGAGTACACGCCGATGTGGATTGGAAAACTGAGAAACAGCATCGTACT 2940

TRINITY_DN4986 GTACTCTTCAACAGAGTACACGCCGATGTGGATTGGAAAACTGAGAAACAGCATCGTACT 2934

************************************************************

TRINITY_DN8321 GAATCGAAGACAGTACCTATCCCATGCGAAGTAGTTTATTCACTAGCGACCTCGATTTTC 3000

TRINITY_DN4986 GAATCGAAGACAGTACCTATCCCATGCGAAGTAGTTTATTCACTAGCGACCTCGATTTTC 2994

************************************************************

TRINITY_DN8321 AAATCTGGTATCGCAGAAGGCCGTGCACCTTTGCGTTTGGATTGGAACGGTTACTGGGCT 3060

TRINITY_DN4986 AAATCTGGTATCGCAGAAGGCCGTGCACCTTTGCGTTTGGATTGGAACGGTTACTGGGCT 3054

************************************************************

TRINITY_DN8321 GGAAGGTGGTCATCAATGCCTAATGGCTCAATTGTAAGCCAGTACGAAACTGACTTAGAG 3120

TRINITY_DN4986 GGAAGGTGGTCATCAATGCCTAATGGCTCAATTGTAAGCCAGTACGAAACTGACTTAGAG 3114

************************************************************

TRINITY_DN8321 TTAAAGCGCTCGCTACCAAAAGAAGCGAAGTACAAGTCGTGCTGGTTTGCAGTGAATGGT 3180

TRINITY_DN4986 TTAAAGCGCTCGCTACCAAAAGAAGCGAAGTACAAGTCGTGCTGGTTTGCAGTGAATGGT 3174

************************************************************

TRINITY_DN8321 CACAGTGATCACAGCTTCTGGGCGAAGCGCAACCCCGAAATTTATGCTACTACAAGCACT 3240

TRINITY_DN4986 CACAGTGATCACAGCTTCTGGGCGAAGCGCAACCCCGAAATTTATGCTACTACAAGCACT 3234

************************************************************

TRINITY_DN8321 AAGTATGAATGGGGCAAAGTGCGCGCTCTGTACGGCTGTGATGTTACTTCATTTCTTCAT 3300

TRINITY_DN4986 AAGTATGAATGGGGCAAAGTGCGCGCTCTGTACGGCTGTGATGTTACTTCATTTCTTCAT 3294

************************************************************

TRINITY_DN8321 GCAGACTTCGCGATGAGCAACTGTGAAGATACCCTGCCTTCCTGTTTCCCTGTAGGCAAG 3360

TRINITY_DN4986 GCAGACTTCGCGATGAGCAACTGTGAAGATACCCTGCCTTCCTGTTTCCCTGTAGGCAAG 3354

************************************************************

TRINITY_DN8321 TTCGCAACAAAGAGCTTTGTCGAAGGCAGTGTGCACAAGTTTAAAGACACTGTGCCGGTA 3420

TRINITY_DN4986 TTCGCAACAAAGAGCTTTGTCGAAGGCAGTGTGCACAAGTTTAAAGACACTGTGCCGGTA 3414

************************************************************

TRINITY_DN8321 TGCTTCGATTATGATGACTTCAACAGTCAGCACTCAATTGCTAGTATGCAAGCAGTCATG 3480

TRINITY_DN4986 TGCTTCGATTATGATGACTTCAACAGTCAGCACTCAATTGCTAGTATGCAAGCAGTCATG 3474

************************************************************

TRINITY_DN8321 CGTGCTTGGATTGATGTCTATAGTACCTTTCTAACTGAGGAACAGAAGATATCAGCTGAG 3540

TRINITY_DN4986 CGTGCTTGGATTGATGTCTATAGTACCTTTCTAACTGAGGAACAGAAGATATCAGCTGAG 3534

************************************************************

TRINITY_DN8321 TGGACGCATGACTCGCTTGCGAATATGAGTGTATGCTTCAACGCGTTGGGTGAGACTGTC 3600

TRINITY_DN4986 TGGACGCATGACTCGCTTGCGAATATGAGTGTATGCTTCAACGCGTTGGGTGAGACTGTC 3594

************************************************************

TRINITY_DN8321 ACTATTGATGGTACTCTCATGAGCGGCTGGCGTCTTACATCTTACATGAACACAGTGCTG 3660

TRINITY_DN4986 ACTATTGATGGTACTCTCATGAGCGGCTGGCGTCTTACATCTTACATGAACACAGTGCTG 3654

************************************************************

TRINITY_DN8321 AATCGTGTCTATCTACTACATGCAGGTTTAGACAAGCTACTTGTATATTCTTTGCATAAT 3720

TRINITY_DN4986 AATCGTGTCTATCTACTACATGCAGGTTTAGACAAGCTACTTGTATATTCTTTGCATAAT 3714

************************************************************

TRINITY_DN8321 GGTGATGACATGTTCGGCGGTGCTCCTAATCTGCACAATGCGCTGCAGCTAATCAAGAAT 3780

TRINITY_DN4986 GGTGATGACATGTTCGGCGGTGCTCCTAATCTGCACAATGCGCTGCAGCTAATCAAGAAT 3774

************************************************************

TRINITY_DN8321 GCTAAAGAACGTGGCATTCGCGCCCAGGTCAGTAAGACTAACCTGGGCACTATTGGCGAA 3840

TRINITY_DN4986 GCTAAAGAACGTGGCATTCGCGCCCAGGTCAGTAAGACTAACCTGGGCACTATTGGCGAA 3834

************************************************************

TRINITY_DN8321 TTCCTTCGCGTCGACACGCGCGCAAAGGATTCACAGATGACGCAGTATCTGGCCCGCGCC 3900

TRINITY_DN4986 TTCCTTCGCGTCGACACGCGCGCAAAGGATTCACAGATGACGCAGTATCTGGCCCGCGCC 3894

************************************************************

TRINITY_DN8321 GTCTCAACGCTGGTTCACGGCCGTGTTGAGGCTGATTCACCCACTGATCTAGTGGCCTTC 3960

TRINITY_DN4986 GTCTCAACGCTGGTTCACGGCCGTGTTGAGGCTGATTCACCCACTGATCTAGTGGCCTTC 3954

************************************************************

TRINITY_DN8321 ATCACCGCTACGATCACACGTGTTGAAGAGGTGAAGTCCCGCGGCGGTAGTGCCACTGTG 4020

TRINITY_DN4986 ATCACCGCTACGATCACACGTGTTGAAGAGGTGAAGTCCCGCGGCGGTAGTGCCACTGTG 4014

************************************************************

TRINITY_DN8321 CTAGACGCATTGTTCAACAAGATTATGGAGTTTGCTTCTAAGTTATTCAGTACTGATCCA 4080

TRINITY_DN4986 CTAGACGCATTGTTCAACAAGATTATGGAGTTTGCTTCTAAGTTATTCAGTACTGATCCA 4074

************************************************************

TRINITY_DN8321 GAAGTAATTGAAGCTATTCTCACAACGCATCCCACTCAAGGTGGTGTTAATAAAGACGCT 4140

TRINITY_DN4986 GAAGTAATTGAAGCTATTCTCACAACGCATCCCACTCAAGGTGGTGTTAATAAAGACGCT 4134

************************************************************

TRINITY_DN8321 CCTGTACGAGCAAAGTACCTGAAGCGGAAGGCACACTCCGGCAAGGATGATATCTACTAC 4200

TRINITY_DN4986 CCTGTACGAGCAAAGTACCTGAAGCGGAAGGCACACTCCGGCAAGGATGATATCTACTAC 4194

************************************************************

TRINITY_DN8321 CATCAGAAGTACTCTATTCTCGGGGACGGAATTAATGATTACATTAATCATGTAAAGCAG 4260

TRINITY_DN4986 CATCAGAAGTACTCTATTCTCGGGGACGGAATTAATGATTACATTAATCATGTAAAGCAG 4254

************************************************************

TRINITY_DN8321 AGATTCCATCTCCGAGAAAGTGAGCTAGATCGCAGTAGACTCCAGCTGAAGGCCTACCAG 4320

TRINITY_DN4986 AGATTCCATCTCCGAGAAAGTGAGCTAGATCGCAGTAGACTCCAGCTGAAGGCCTACCAG 4314

************************************************************

TRINITY_DN8321 TCTTTGGAACGCGATCTTGTTAGTTACGAGACCGGAATCGAAACTAATGAGAGAATCGCA 4380

TRINITY_DN4986 TCTTTGGAACGCGATCTTGTTAGTTACGAGACCGGAATCGAAACTAATGAGAGAATCGCA 4374

************************************************************

TRINITY_DN8321 ATCTACAGAGGTCTAAGTGGTGCATGGAAGGGCACAGGTTTTGAAGCACCTATTGCTAAA 4440

TRINITY_DN4986 ATCTACAGAGGTCTAAGTGGTGCATGGAAGGGCACAGGTTTTGAAGCACCTATTGCTAAA 4434

************************************************************

TRINITY_DN8321 GTCAGATCGATGGGTCTCATTGCAGCCAAGCAACTGAGAGTGCTGACATCTACTTTAGCT 4500

TRINITY_DN4986 GTCAGATCGATGGGTCTCATTGCAGCCAAGCAACTGAGAGTGCTGACATCTACTTTAGCT 4494

************************************************************

TRINITY_DN8321 AGGATGATTCAGAATGCTGATGACCCTATTACTTTCATGCGAGTAGTCACTTAGACCTCT 4560

TRINITY_DN4986 AGGATGATTCAGAATGCTGATGACCCTATTACTTTCATGCGAGTAGTCACTTAGACCTCT 4554

************************************************************

TRINITY_DN8321 GTATAAATCGTTCTGCAGCTTTAGGCTGGCAGAACGCCGTGCGTTATTATATA 4613

TRINITY_DN4986 GTATAAATCGTTCTGCAGCTTTAGGCTGGCAGAACGCCGTGCGTTAT------ 4601

***********************************************

Blastn against the transcriptome of the virus-cured *M. sympodialis* SEC494 strain finds one hit:

TRINITY_DN4775_c0_g1_i1 len=309 path=[287:0-308] [-1, 287, -2] Score 508 E value 1e-143

GGCGTCCTGACGAACGCGCCGAAGCTGCTGTCGTTTGCGACGCAGTACAGCGTGCAGCGCTCGCGCCGCGCCGAGGACCACGACCAGGCGGCGCCGAACATGACGCAGCTCGCGACGCTGCAGTACCGCCCCGACCTGGTCATCGTGCTGAACCCCACAGAAAATGAGAATGCACTGCGCGAGGCGACCCAATCGAACGTGCCGACCATGGCCATCGTGGATACCAACGTCGACCCACGCGCCGTGACCTACGCGATTCCCGCCAACGAC

Blastx results: MSYG_4110, accession SHO79760, similar to *S. cerevisiae* protein *MRP4* (Mitochondrial ribosomal protein of the small subunit) [*Malassezia sympodialis* ATCC 42132]
