# Supplementary material for: A Novel Mycovirus Evokes Transcriptional Rewiring in the Fungus Malassezia and Stimulates Beta Interferon Production in Macrophages
Source: mBio. 2020 Sep 1;11(5):e01534-20. doi: 10.1128/mBio.01534-20 (PMC7468202; doi:10.1128/mBio.01534-20)
Supplement: TEXT S2 [file mBio.01534-20-s0002.docx]

>TA-cloning Small fragment (used as query for blastn)

TGTAAAGTTTTGTTGCATAGTGTGCCTTGGCACCCGAGAATTCCATGATATTTACGGCTTCTGTTTAATTATGTTTTCTAGAAATATAATTATCTTTACTACTGTTGCTTCTGCCTTCGCCATAATAGCACGTGCAGACGTCAACACCACCAACATTCCAACCACCGATTTACCAGCTTGTTGGATACATGGCGATAATAACGCCACTGCCACACTATGCAACAAAGCTTTACTAATAAAGCCAGAGAGTGGTGATTATTATTGTAGAAATGCCGTAAACACATTGAATAACACACTAACAAGCAGTCCTGACTTTAGATGTTATGACCGTCGTGACGATGACAATAACAGTGCTACCATGACTGCCTTCAATT

Blastn against the transcriptome of the virus-infected *M. sympodialis* KS012 strain produced two hits:

1) TRINITY_DN7179_c0_g1_i1 len=1546 path=[1548:0-1545] [-1, 1548, -2] Score 595 **E-value 3e-170** 2) TRINITY_DN6053_c1_g1_i1 len=1479 path=[1458:0-1478] [-1, 1458, -2] Score 582 **E- value 2e-166**

**1) HIT 1**

**>TRINITY_DN7179_c0_g1_i1 len=1546 path=[1548:0-1545] [-1, 1548, -2]** (displayed in reverse complement orientation)

In red is shown the matching sequence following blastn

CCTGACTTTAGATGTTATGACCGCTTGTTAGTGTGTCATTCAATGTATTTACGGCTTCTGTTTAATTATGTTTTCTAGAAATATAATTATCTTTACTACTGTTGCTTCTGCCTTCGCCATAATAGCACGTGCAGACGTCAACACCACCAACATTCCAACCACCGATTTACCAGCTTGTTGGATACATGGCGATAATAACGCCACTGCCACACTATGCAACAAAGCTTTACTAATAAAGCCAGAGAGTGGTGATTATTATTGTAGAAATGCCGTAAACACATTGAATGACACACTAACAAGCAGTCCTGACTTTAGATGTTATGACCGTCGTGACGATGACAATAACAGTGCTACCATGACTGCCTTCAGTTTGCCCTTAAGTGTAAGTATATTAATTATTAATATTTTATACTATATATATTAGGAACTCGCTACCTTTAAGAACACTACTCCGCTGTCTGCTGCTGTATTTCACAGGACCCATTACTTCAGCAGTTTTTCCGTCACCAGCTTGAGGTTTAATACTTAACCATTGTACTCTAGGTGAATACTGGGATCTTCCCTTGGAACGACAACATTGCAAGGGACATCATCTCCATCACACTGAGTGCATTGACCATTGTAACTGTCAGCAGGTGCTGAAGCCACCAATCTAGCTTTCGAGCCAGGTGGGATAGAGTATTCTCGACCTACAGTAGTCGATCTCGATTCCTCATAGCTCGTTCCAACTTCGGTGTTGACTGACACTATAAAGTCTGACCCAACAGACACTGACACTGTCATACCAACAGTCACCGACTGTTCTTCATCAACATTGATCTTACAATCATCACTCGACCCCGTGCAATCAGTAAAGCTGTTTGGCACATCAACTGATGGTGTATTGGTTCCTTCAGCTGGTTTATTATCCCATTTGAAAGCTTTGCAGTTCTCTGAGCGTTTGCCTAGCTTATAACGCCCATTGTTAGCATCAGTATCAATCCACTGAGTGTTACCATCAATCTTATTCTCACCAACGTTCTTGACTGTATAGCCATTACCACCTTCGAACTTGACAGGGATACAGCGGCCATCACCAGGATCAAAAGTCGCGAGCTTATCACCCTCACCCTTCTCCGACGGTGGACAAGTGTGGTCTACGAGCAAATATGCTTGAGTACCCATCATTCCACCAGATGTTTGAATCGAGTTAAAAGGATTTGGAATTGCAGACACATCGTTACAGTTCCATTCGGATCCATCGAAATCATCGCCATAGTGTACGCAGTTAGTGAAATGGCGCGGGTTGTCATTGCAACCATTGTCATCAAAAACTGTGAAAGAGAACATGGGCCACGCCTTATCTTCCCAAGCTGTGCATTGTCCTTGGGCATCATCACGACTGATTTTCATGCTACCATTCGAGGTTAATACGGTTGTGTTGTGCTCGACAAGTCTTGTTTTAAGTGCCAGAGCGGCACCACAACAGACAGAAATGTAGACAAAAAACAGTTTGTTATACATAGTTAAGTTATATAAATTATCAGAGCGGCACCACAACAGACAG

**2) HIT 2**

**>TRINITY_DN6052_c1_g1_i1 len=1479 path=[1458:0-1478] [-1, 1458, -2]**

In red is shown the matching sequence following blastn

CACGGCTTCTGTTTAATTATGTTTTCTAGAAATATAATTATCTTTACTACTGTTGCTTCTGCCTTCGCCATAATAGCACGTGCAGACGTCAACACCACCAACATTCCAACCACCGATTTACCAGCTTGTTGGATACATGGCGATAATAACGCCACTGCCACACTATGCAACAAAGCTTTACTAATAAAGCCAGAGAGTGGTGATTATTATTGTAGAAATGCCGTAAACACATTGAATGACACACTAACAAGCAGTCCTGACTTTAGATGTTATGACCGTCGTGACGATGACAATAACAGTGCTACCATGACTGCCTTCAGTTTGCCCTTAAGTGTAAGTATATTAATTATTAATATTTTATACTATATATATTAGGAACTCGCTACCTTTAAGAACACTACTCCGCTGTCTGCTGCTGTATTTCACAGGACCCATTACTTCAGCAGTTTTTCCGTCACCAGCTTGAGGTTTAATACTTAACCATTGTACTCTAGGTGAATACTGGGATCTTCCCTTGGAACGACAACATTGCAAGGGACATCATCTCCATCACACTGAGTGCATTGACCATTGTAACTGTCAGCAGGTGCTGAAGCCACCAATCTAGCTTTCGAGCCAGGTGGGATAGAGTATTCTCGACCTACAGTAGTCGATCTCGATTCCTCATAGCTCGTTCCAACTTCGGTGTTGACTGACACTATAAAGTCTGACCCAACAGACACTGACACTGTCATACCAACAGTCACCGACTGTTCTTCATCAACATTGATCTTACAATCATCACTCGACCCCGTGCAATCAGTAAAGCTGTTTGGCACATCAACTGATGGTGTATTGGTTCCTTCAGCTGGTTTATTATCCCATTTGAAAGCTTTGCAGTTCTCTGAGCGTTTGCCTAGCTTATAACGCCCATTGTTAGCATCAGTATCAATCCACTGAGTGTTACCATCAATCTTATTCTCACCAACGTTCTTGACTGTATAGCCATTACCACCTTCGAACTTGACAGGGATACAGCGGCCATCACCAGGATCAAAAGTCGCGAGCTTATCACCCTCACCCTTCTCCGACGGTGGACAAGTGTGGTCTACGAGCAAATATGCTTGAGTACCCATCATTCCACCAGATGTTTGAATCGAGTTAAAAGGATTTGGAATTGCAGACACATCGTTACAGTTCCATTCGGATCCATCGAAATCATCGCCATAGTGTACGCAGTTAGTGAAATGGCGCGGGTTGTCATTGCAACCATTGTCATCAAAAACTGTGAAAGAGAACATGGGCCACGCCTTATCTTCCCAAGCTGTGCATTGTCCTTGGGCATCATCACGACTGATTTTCATGCTACCATTCGAGGTTAATACGGTTGTGTTGTGCTCGACAAGTCTTGTTTTAAGTGCCAGAGCGGCACCACAACAGACAGAAATGTAGACAAAAAACAGTTTGTTATACATAGTTAAGTTATATAAATTATCGGGG

CLUSTAL O(1.2.4) multiple sequence alignment

TRINITY_DN7179_c0_g1_i1 CCTGACTTTAGATGTTATGACCGCTTGTTAGTGTGTCATTCAATGTATTTACGGCTTCTG 60

TRINITY_DN6052_c1_g1_i1 -------------------------------------------------CACGGCTTCTG 11

**********

TRINITY_DN7179_c0_g1_i1 TTTAATTATGTTTTCTAGAAATATAATTATCTTTACTACTGTTGCTTCTGCCTTCGCCAT 120

TRINITY_DN6052_c1_g1_i1 TTTAATTATGTTTTCTAGAAATATAATTATCTTTACTACTGTTGCTTCTGCCTTCGCCAT 71

************************************************************

TRINITY_DN7179_c0_g1_i1 AATAGCACGTGCAGACGTCAACACCACCAACATTCCAACCACCGATTTACCAGCTTGTTG 180

TRINITY_DN6052_c1_g1_i1 AATAGCACGTGCAGACGTCAACACCACCAACATTCCAACCACCGATTTACCAGCTTGTTG 131

************************************************************

TRINITY_DN7179_c0_g1_i1 GATACATGGCGATAATAACGCCACTGCCACACTATGCAACAAAGCTTTACTAATAAAGCC 240

TRINITY_DN6052_c1_g1_i1 GATACATGGCGATAATAACGCCACTGCCACACTATGCAACAAAGCTTTACTAATAAAGCC 191

************************************************************

TRINITY_DN7179_c0_g1_i1 AGAGAGTGGTGATTATTATTGTAGAAATGCCGTAAACACATTGAATGACACACTAACAAG 300

TRINITY_DN6052_c1_g1_i1 AGAGAGTGGTGATTATTATTGTAGAAATGCCGTAAACACATTGAATGACACACTAACAAG 251

************************************************************

TRINITY_DN7179_c0_g1_i1 CAGTCCTGACTTTAGATGTTATGACCGTCGTGACGATGACAATAACAGTGCTACCATGAC 360

TRINITY_DN6052_c1_g1_i1 CAGTCCTGACTTTAGATGTTATGACCGTCGTGACGATGACAATAACAGTGCTACCATGAC 311

************************************************************

TRINITY_DN7179_c0_g1_i1 TGCCTTCAGTTTGCCCTTAAGTGTAAGTATATTAATTATTAATATTTTATACTATATATA 420

TRINITY_DN6052_c1_g1_i1 TGCCTTCAGTTTGCCCTTAAGTGTAAGTATATTAATTATTAATATTTTATACTATATATA 371

************************************************************

TRINITY_DN7179_c0_g1_i1 TTAGGAACTCGCTACCTTTAAGAACACTACTCCGCTGTCTGCTGCTGTATTTCACAGGAC 480

TRINITY_DN6052_c1_g1_i1 TTAGGAACTCGCTACCTTTAAGAACACTACTCCGCTGTCTGCTGCTGTATTTCACAGGAC 431

************************************************************

TRINITY_DN7179_c0_g1_i1 CCATTACTTCAGCAGTTTTTCCGTCACCAGCTTGAGGTTTAATACTTAACCATTGTACTC 540

TRINITY_DN6052_c1_g1_i1 CCATTACTTCAGCAGTTTTTCCGTCACCAGCTTGAGGTTTAATACTTAACCATTGTACTC 491

************************************************************

TRINITY_DN7179_c0_g1_i1 TAGGTGAATACTGGGATCTTCCCTTGGAACGACAACATTGCAAGGGACATCATCTCCATC 600

TRINITY_DN6052_c1_g1_i1 TAGGTGAATACTGGGATCTTCCCTTGGAACGACAACATTGCAAGGGACATCATCTCCATC 551

************************************************************

TRINITY_DN7179_c0_g1_i1 ACACTGAGTGCATTGACCATTGTAACTGTCAGCAGGTGCTGAAGCCACCAATCTAGCTTT 660

TRINITY_DN6052_c1_g1_i1 ACACTGAGTGCATTGACCATTGTAACTGTCAGCAGGTGCTGAAGCCACCAATCTAGCTTT 611

************************************************************

TRINITY_DN7179_c0_g1_i1 CGAGCCAGGTGGGATAGAGTATTCTCGACCTACAGTAGTCGATCTCGATTCCTCATAGCT 720

TRINITY_DN6052_c1_g1_i1 CGAGCCAGGTGGGATAGAGTATTCTCGACCTACAGTAGTCGATCTCGATTCCTCATAGCT 671

************************************************************

TRINITY_DN7179_c0_g1_i1 CGTTCCAACTTCGGTGTTGACTGACACTATAAAGTCTGACCCAACAGACACTGACACTGT 780

TRINITY_DN6052_c1_g1_i1 CGTTCCAACTTCGGTGTTGACTGACACTATAAAGTCTGACCCAACAGACACTGACACTGT 731

************************************************************

TRINITY_DN7179_c0_g1_i1 CATACCAACAGTCACCGACTGTTCTTCATCAACATTGATCTTACAATCATCACTCGACCC 840

TRINITY_DN6052_c1_g1_i1 CATACCAACAGTCACCGACTGTTCTTCATCAACATTGATCTTACAATCATCACTCGACCC 791

************************************************************

TRINITY_DN7179_c0_g1_i1 CGTGCAATCAGTAAAGCTGTTTGGCACATCAACTGATGGTGTATTGGTTCCTTCAGCTGG 900

TRINITY_DN6052_c1_g1_i1 CGTGCAATCAGTAAAGCTGTTTGGCACATCAACTGATGGTGTATTGGTTCCTTCAGCTGG 851

************************************************************

TRINITY_DN7179_c0_g1_i1 TTTATTATCCCATTTGAAAGCTTTGCAGTTCTCTGAGCGTTTGCCTAGCTTATAACGCCC 960

TRINITY_DN6052_c1_g1_i1 TTTATTATCCCATTTGAAAGCTTTGCAGTTCTCTGAGCGTTTGCCTAGCTTATAACGCCC 911

************************************************************

TRINITY_DN7179_c0_g1_i1 ATTGTTAGCATCAGTATCAATCCACTGAGTGTTACCATCAATCTTATTCTCACCAACGTT 1020

TRINITY_DN6052_c1_g1_i1 ATTGTTAGCATCAGTATCAATCCACTGAGTGTTACCATCAATCTTATTCTCACCAACGTT 971

************************************************************

TRINITY_DN7179_c0_g1_i1 CTTGACTGTATAGCCATTACCACCTTCGAACTTGACAGGGATACAGCGGCCATCACCAGG 1080

TRINITY_DN6052_c1_g1_i1 CTTGACTGTATAGCCATTACCACCTTCGAACTTGACAGGGATACAGCGGCCATCACCAGG 1031

************************************************************

TRINITY_DN7179_c0_g1_i1 ATCAAAAGTCGCGAGCTTATCACCCTCACCCTTCTCCGACGGTGGACAAGTGTGGTCTAC 1140

TRINITY_DN6052_c1_g1_i1 ATCAAAAGTCGCGAGCTTATCACCCTCACCCTTCTCCGACGGTGGACAAGTGTGGTCTAC 1091

************************************************************

TRINITY_DN7179_c0_g1_i1 GAGCAAATATGCTTGAGTACCCATCATTCCACCAGATGTTTGAATCGAGTTAAAAGGATT 1200

TRINITY_DN6052_c1_g1_i1 GAGCAAATATGCTTGAGTACCCATCATTCCACCAGATGTTTGAATCGAGTTAAAAGGATT 1151

************************************************************

TRINITY_DN7179_c0_g1_i1 TGGAATTGCAGACACATCGTTACAGTTCCATTCGGATCCATCGAAATCATCGCCATAGTG 1260

TRINITY_DN6052_c1_g1_i1 TGGAATTGCAGACACATCGTTACAGTTCCATTCGGATCCATCGAAATCATCGCCATAGTG 1211

************************************************************

TRINITY_DN7179_c0_g1_i1 TACGCAGTTAGTGAAATGGCGCGGGTTGTCATTGCAACCATTGTCATCAAAAACTGTGAA 1320

TRINITY_DN6052_c1_g1_i1 TACGCAGTTAGTGAAATGGCGCGGGTTGTCATTGCAACCATTGTCATCAAAAACTGTGAA 1271

************************************************************

TRINITY_DN7179_c0_g1_i1 AGAGAACATGGGCCACGCCTTATCTTCCCAAGCTGTGCATTGTCCTTGGGCATCATCACG 1380

TRINITY_DN6052_c1_g1_i1 AGAGAACATGGGCCACGCCTTATCTTCCCAAGCTGTGCATTGTCCTTGGGCATCATCACG 1331

************************************************************

TRINITY_DN7179_c0_g1_i1 ACTGATTTTCATGCTACCATTCGAGGTTAATACGGTTGTGTTGTGCTCGACAAGTCTTGT 1440

TRINITY_DN6052_c1_g1_i1 ACTGATTTTCATGCTACCATTCGAGGTTAATACGGTTGTGTTGTGCTCGACAAGTCTTGT 1391

************************************************************

TRINITY_DN7179_c0_g1_i1 TTTAAGTGCCAGAGCGGCACCACAACAGACAGAAATGTAGACAAAAAACAGTTTGTTATA 1500

TRINITY_DN6052_c1_g1_i1 TTTAAGTGCCAGAGCGGCACCACAACAGACAGAAATGTAGACAAAAAACAGTTTGTTATA 1451

************************************************************

TRINITY_DN7179_c0_g1_i1 CATAGTTAAGTTATATAAATTATCAGAGCGGCACCACAACAGACAG 1546

TRINITY_DN6052_c1_g1_i1 CATAGTTAAGTTATATAAATTATCGGGG------------------ 1479

************************ * *
